# Effect of long-term exposure of SH-SY5Y cells to morphine: a whole cell proteomic analysis

**DOI:** 10.1186/1477-5956-4-23

**Published:** 2006-12-21

**Authors:** Jérémie Neasta, Sandrine Uttenweiler-Joseph, Karima Chaoui, Bernard Monsarrat, Jean-Claude Meunier, Lionel Moulédous

**Affiliations:** 1 Unité *Mécanismes d'action des substances opioïdes*, Institut de Pharmacologie et de Biologie Structurale, Centre National de la Recherche Scientifique UMR 5089, 205 route de Narbonne, 31077 Toulouse cedex 04, France; 2 Unité *Spectrométrie de masse et structure des biomolécules*, Institut de Pharmacologie et de Biologie Structurale, Centre National de la Recherche Scientifique UMR 5089, 205 route de Narbonne, 31077 Toulouse cedex 04, France

## Abstract

**Background:**

Opiate addiction reflects plastic changes that endurably alter synaptic transmission within relevant neuronal circuits. The biochemical mechanisms of these adaptations remain largely unknown and proteomics-based approaches could lead to a broad characterization of the molecular events underlying adaptations to chronic drug exposure.

**Results:**

Thus, we have started proteomic analyses of the effects of chronic morphine exposure in a recombinant human neuroblastoma SH-SY5Y clone that stably overexpresses the μ-opioid receptor. Cells were treated with morphine for 6, 24 and 72 hours, the proteins were separated by 2-D gel electrophoresis and stained with Coomassie blue, and the protein map was compared with that obtained from untreated cells. Spots showing a statistically significant variation were selected for identification using mass spectrometric analyses.

**Conclusion:**

A total of 45 proteins were identified, including proteins involved in cellular metabolism, cytoskeleton organization, vesicular trafficking, transcriptional and translational regulation, and cell signaling.

## Background

Opiate addiction, a pathological form of learning and memory associated with repeated drug use or administration, reflects neuronal adaptive/plastic changes that endurably alter synaptic transmission within relevant circuits in the central nervous system [1-4]. The biochemical mechanisms underlying the functional and structural adaptations to chronic opiate exposure remain largely unknown. Elucidating them in details is important, as this is expected to reveal novel pharmacological strategies for preventing formation and/or expression of dependence, with potential benefits for the treatment of chronic pain and addiction.

The biochemical mechanisms of drug dependence have begun to be examined globally by using DNA microarray- and/or proteomics-based approaches. Thus, DNA microarray-based approaches have been used in order to study gene expression induced by drugs of abuse [5], including opiates [6,7], but interpretation of the results is limited – mRNA levels do not necessarily reflect proteins levels [8] – and no information about post-transcriptionally modified proteins is provided. In principle, proteomics-based approaches could lead to a much broader characterization of the molecular events underlying drug dependence. Yet, the successful application of differential proteomics to identify drug-induced protein changes in the central nervous system represent a technical challenge because of its cellular heterogeneity [9].

Obviously, sample heterogeneity is much less of a problem in cultured clonal cell lines than in nerve tissue. Thus, we have started proteomic analyses of the effects of chronic morphine exposure in a recombinant human neuroblastoma SH-SY5Y clone that stably overexpresses the μ-opioid (MOP) receptor. Wild-type SH-SY5Y cells express low levels of MOP receptor, and even lower (3- to 4-fold) levels of delta opioid (DOP) receptor [10], and are only poorly responsive to both acute and long-term morphine treatment [11]. In marked contrast, in MOP receptor-overexpressing cells, acute morphine is much more potent and efficacious in inhibiting forskolin-elicited production of cAMP, and chronic morphine induces a higher degree of adenylate cyclase sensitization, a hallmark of opiate dependence, than in the parent (wild type) cells [11]. The dramatically increased responsiveness of MOP-overexpressing over wild-type cells is an indication that the observed effects are MOP receptor- rather than DOP receptor-mediated. The cells were treated with morphine for 6, 24 and 72 hours, the proteins were separated by 2-D electrophoresis (2-DE) and stained with colloidal Coomassie blue, and the protein map was compared with that obtained from untreated cells. Spots showing a statistically significant variation were selected for identification using a combination of MALDI-TOF MS (matrix-assisted laser desorption/ionization time of flight mass spectrometry) and nanoLC-ESI-Q-TOF MS/MS (liquid-chromatography electrospray ionization quadrupole time of flight) analyses. A total of 45 proteins were found to have varied in abundance in the course of long-term exposure to morphine, including proteins involved in cellular metabolism, cytoskeleton organization, vesicular trafficking, transcriptional and translational regulation, and cell signaling.

## Results

**Figure 1:**
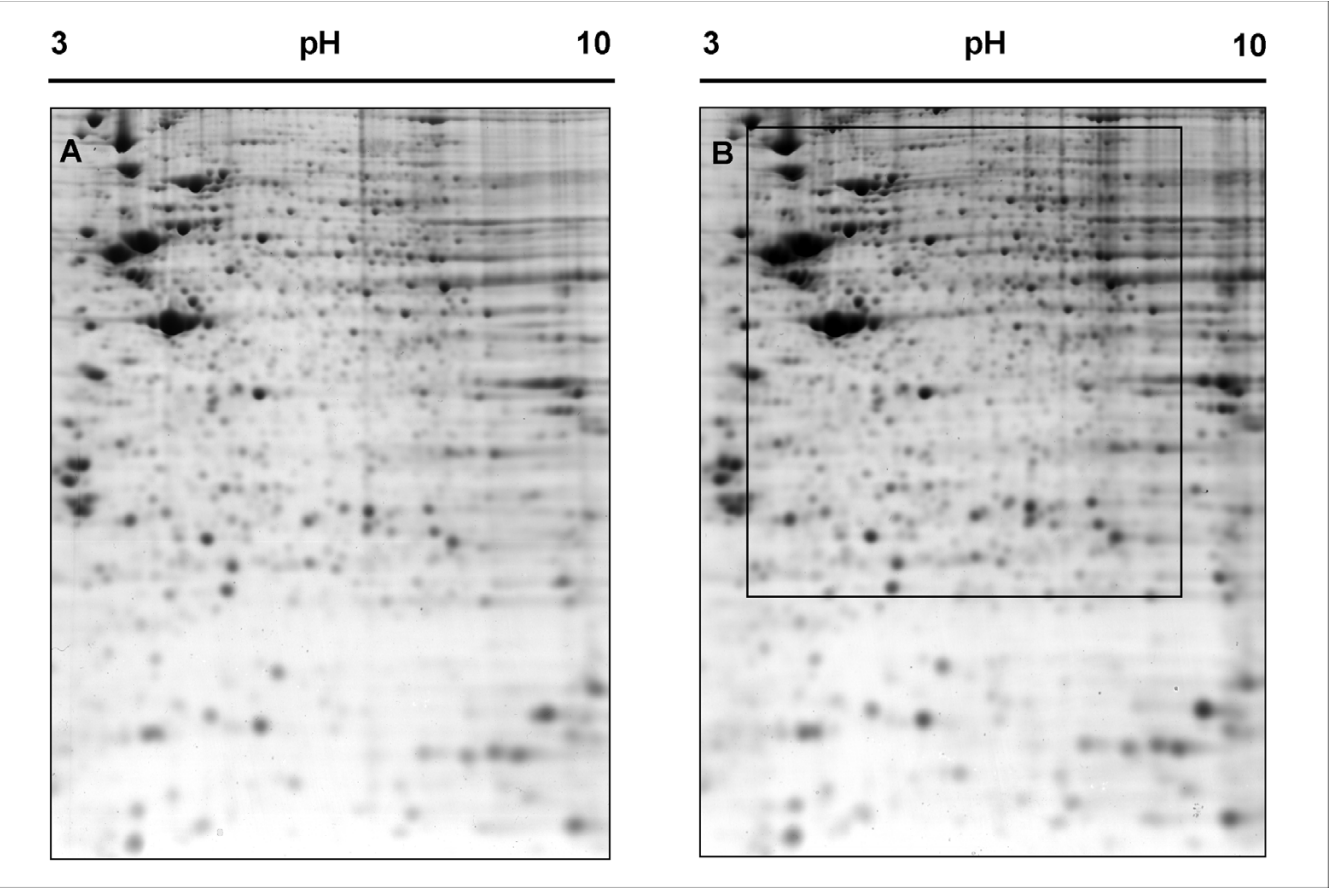
**2-DE pattern of untreated (A) and 6 h morphine-treated (B) SH-SY5Y cells**. Sample were resolved by 2-DE on non-linear pH 3–10 IPG strips followed by separation on a 12% SDS-PAGE gel in the second dimension. Proteins were visualized by colloidal coomassie staining. The box in 1B delineates the close-up presented on figure 2.

Figure 1 shows a representative bi-dimensional map of the proteome of untreated (control) neuroblastoma SH-SY5Y cells. Colloidal Coomassie blue protein staining, followed by automatic feature detection and manual editing enabled visualization of about 950 individual spots on a 2-D gel.

**Figure 2:**
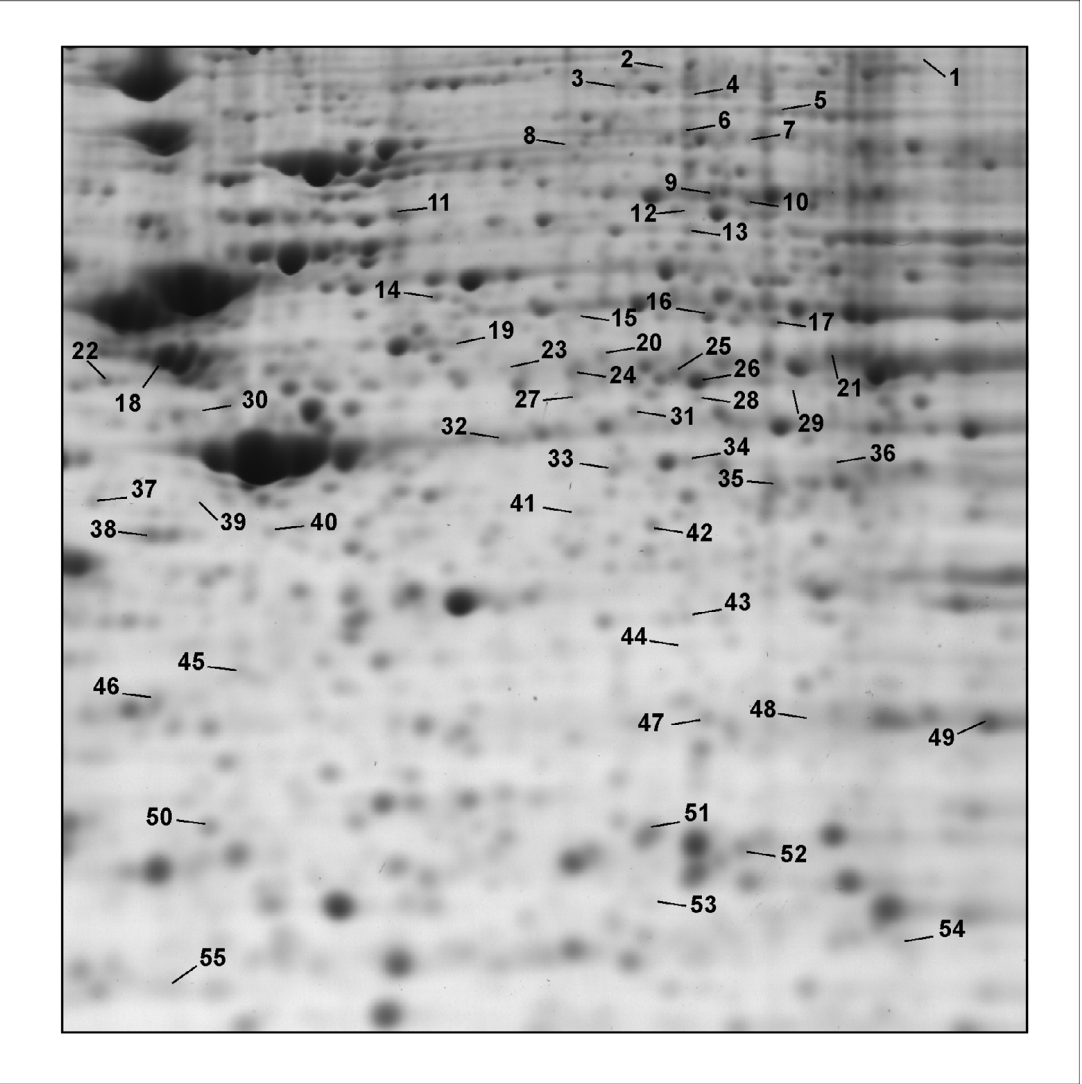
**Close-up from a representative 2-D gel showing the spots whose abundance in SH-SY5Y cells is regulated after chronic morphine treatment**. Spots numbers refer to numbers on Tables 1, 2 and 3 where quantitative analysis and mass spectrometric data are presented.

The protein map of neuroblastoma cells having been treated with 1 μM morphine for 6 hours was compared with that of untreated cells. A 6 h treatment with morphine was selected since such treatment was previously shown to elicit nearly maximum sensitization of adenylate cyclase, an index of opiate dependence, in these cells [11]. As shown on Figure 1, the protein pattern from 6 h-treated cells was globally very similar to that of control cells. After image analysis, the abundance of each spot was estimated relative to the abundance of all spots in the gel (see Methods). Four control gels were matched with four 6 h morphine-treatment gels and the spots showing a statistically significant variation in relative abundance (Student t test, p < 0.05) were selected for identification by mass spectrometry. Eighteen spots displayed such significant variation: 11 spots were upregulated by a factor 1.22 to 2.03 following morphine treatment, while 7 spots were downregulated by a factor 0.35 to 0.75. These spots were cut from a gel, digested by trypsin and analyzed by peptide mass fingerprinting using MALDI-TOF mass spectrometry. When peptide mass fingerprinting data was not sufficient for confident identification, trypsin digests were also analyzed using nanoLC-ESI-MS/MS. The combination of these two methods enabled the unambiguous identification of 18 proteins whose name, accession number, change in relative abundance and MS data are summarized on Table 1. Spot numbers in this table refer to the ones in Figure 2, which depicts the location of every spot that was identified in this study on a representative 2-D gel. Spot 11 could not be identified while spot 24 contained a mixture of 2 proteins.

**Table 1:** Proteins whose abundance in SH-SY5Y cells is regulated after 6 h of morphine treatment

**Spot #**	**Protein**	**Accession # (a)**	**MW (Da)**	**Variation (% of control) (b)**	**MALDI peptides**	**% coverage**	**MS/MS peptides (c)**
2	Elongation factor G 1	Q96RP9	83506	42 ± 11	13	16	
3	Ezrin	P15311	69268	165 ± 26	10	15	
5	Lamin A/C	P02545	74139	203 ± 21	18	32	
9	Bifunctional purine biosynthesis protein	P31939	65088	122 ± 16	14	30	
11	no identification			52 ± 10			
14	Vacuolar ATP synthase subunit B, brain isoform	P21281	56501	151 ± 17	18	54	
15	Rho-GTPase-activating protein 1	Q07960	50461	166 ± 44			4
17	Septin-11	Q9NVA2	49267	141 ± 19	13	37	
18	ATP synthase beta chain	P06576	56560	35 ± 9	10	48	
24	Rab GDP dissociation inhibitor beta Mitochondrial-processing peptidase beta subunit	P50395 O75439	50663 55072	136 ± 16	14 12	36 25	
27	Synaptic vesicle membrane protein VAT-1 homolog	Q99536	41920	141 ± 17	7	31	
28	Adenylosuccinate synthetase 2	P30520	50097	147 ± 14			5
36	40 kDa peptidyl-prolyl cis-trans isomerase	Q08752	40632	174 ± 42	12	26	
44	Malate dehydrogenase, cytoplasmic	P40925	36295	50 ± 9	11	32	
49	Guanine nucleotide binding protein beta subunit 2-like 1	P63244	35077	75 ± 15	15	69	
52	Platelet-activating factor acetylhydrolase IB gamma	Q15102	25734	70 ± 14			5
53	Triosephosphate isomerase	P60174	26538	186 ± 60	13	65	
54	Ras-related protein Rab-7	P51149	23490	56 ± 13	13	65	

^a^Swiss-Prot primary accession number

^b^Mean ± S.D., control refers to untreated cells.

^c^Only peptides with a statistically significant Mascot score (p < 0.05) were considered

We next examined the proteome effects of exposing the neuroblastoma cells to morphine (1 μM) for longer periods of time, *i.e*. 24 and 72 hours. Twenty one spots were found to display a significant variation in relative abundance after a 24 h morphine treatment: 11 spots were upregulated by a factor 1.18 to 1.84, whilst 10 spots were downregulated by a factor 0.45 to 0.84. Among these, 18 spots could be attributed to one protein, and 3 spots contained a mixture of 2 proteins (Table 2). 2 spots with similar molecular weights but different pIs contained the same protein (α-enolase). The relative volume of these 2 spots varied in opposite direction after morphine treatment suggesting a change in post-translational modification, possibly phosphorylation. Twenty-five spots displayed a significant variation in relative abundance after a 72 h morphine treatment: 5 spots were upregulated by a factor 1.1 to 1.79, whilst 20 spots were downregulated by a factor of 0.45 to 0.84. Among these, 21 could be attributed to one protein, 2 contained a mixture of 2 proteins, and 2 could not be identified (Table 3).

**Table 2:** Proteins whose abundance in SH-SY5Y cells is regulated after 24 h of morphine treatment

**Spot #**	**Protein**	**Accession # (a)**	**MW (Da)**	**Variation (% of control) (b)**	**MALDI peptides**	**% coverage**	**MS/MS peptides (c)**
1	ATP-dependent RNA helicase DDX1	Q92499	82432	54 ± 10	27	32	
7	Glycerol-3-phosphate dehydrogenase, mitochondrial	P43304	80815	75 ± 7	15	22	
9	Bifunctional purine biosynthesis protein	P31939	65088	118 ± 11	14	30	
14	Vacuolar ATP synthase subunit B, brain isoform	P21281	56501	131 ± 13	18	54	
16	RuvB-like 1	Q9Y265	50228	144 ± 32	16	66	
19	4-trimethylaminobutyraldehyde dehydrogenase WD-repeat protein 12	P49189 Q9GZL7	53802 48190	145 ± 24	15 12	32 25	
20	Alpha-enolase	P06733	47038	45 ± 15	18	60	
21	Alpha-enolase	P06733	47038	184 ± 53			6
22	Histone acetyltransferase type B subunit 2	Q16576	47820	145 ± 65	10	30	
30	Heterogeneous nuclear riboprotein F Eucaryotic translation initiation factor 3 subunit 5	O00303	45853 37654	181 ± 60	12 7	42 32	
32	Beta-succinyl CoA synthetase	Q7Z503	43611	74 ± 10	15	35	
33	Galactokinase	P51570	42272	58 ± 9	15	43	
35	Acyl-CoA hydrolase	O00154	41796	166 ± 33	11	44	
37	Arsenical pump-driving ATPase	O43681	38793	167 ± 29	8	26	
38	Serine-threonine kinase receptor associated protein	Q9Y3F4	38756	164 ± 31	14	48	
41	Protein phosphatase 2A, regulatory subunit B' Calponin-3	Q15257 Q15417	40682 36561	65 ± 8	10 7	30 30	
42	Biliverdin reductase A	P53004	33428	84 ± 6	12	44	
44	Malate dehydrogenase, cytoplasmic	P40925	36295	49 ± 25	11	32	
46	Annexin A5	P08758	35806	152 ± 41	18	70	
51	Endoplasmic reticulum protein ERp29	P30040	28993	76 ± 14	8	46	
54	Ras-related protein Rab-7	P51149	23490	58 ± 12	13	65	

^a^Swiss-Prot primary accession number

^b^Mean ± S.D., control refers to untreated cells.

^c^Only peptides with a statistically significant Mascot score (p < 0.05) were considered

**Table 3:** Proteins whose abundance in SH-SY5Y cells is regulated after 72 h of morphine treatment

**Spot #**	**Protein**	**Accession # (a)**	**MW (Da)**	**Variation (% of control) (b)**	**MALDI peptides**	**% coverage**	**MS/MS peptides (c)**
4	Radixin	P35241	68564	50 ± 17			4
6	no identification			48 ± 23			
8	Heat shock cognate 71 kDa protein	P11142	70898	172 ± 48	11	23	
10	Lamin A/C	P02545	74139	78 ± 15	22	30	
12	T-complex protein 1, zeta subunit	P40227	57893	57 ± 17	15	29	
13	Dihydropyrimidinase-related	Q14195	61963	57 ± 21	13	40	
	protein 3						
17	Septin-11	Q9NVA2	49267	110 ± 3	13	37	
23	Rab GDP dissociation inhibitor beta	P50395	50663	179 ± 61	11	34	
24	Rab GDP dissociation inhibitor beta Mitochondrial-processing peptidase beta subunit	P50395 O75439	50663 55072	114 ± 6	14 12	36 25	
25	Proliferation-associated protein 2G4	Q9UQ80	43787	54 ± 20	15	44	
26	Elongation factor 1-gamma	P26641	49988	89 ± 5	16	36	
28	Adenylosuccinate synthetase 2	P30520	50097	73 ± 16			5
29	Elongation factor 1-gamma tRNA-nucleotidyltransferase 1	P26641 Q96Q11	49988 50340	51 ± 27	11 9	24 19	
31	Ornithine aminotransferase.	P04181	48535	84 ± 3	14	37	
34	Septin-2	Q15019	41487	72 ± 15	12	36	
39	Ubiquitin-like 1 activating enzyme E1A	Q9UBE0	38450	45 ± 14	11	35	
40	Guanine nucleotide binding protein, alpha inhibiting activity polypeptide 2	Q96C71	40493	65 ± 8	9	37	
43	3-mercaptopyruvate sulfurtransferase	P25325	33047	69 ± 9			6
44	Malate dehydrogenase, cytoplasmic	P40925	36295	73 ± 16	11	32	
45	Alpha-soluble NSF attachment protein	P54920	33247	45 ± 12	8	45	
47	Nuclear protein Hcc-1	P82979	23540	77 ± 9	9	41	
48	no identification			49 ± 32			
49	Guanine nucleotide binding protein beta subunit 2-like 1	P63244	35077	74 ± 9	15	69	
50	Proteasome subunit alpha type 3	P25788	28302	73 ± 13	8	46	
55	Proteasome subunit beta type 6	P28072	25358	143 ± 11	7	23	

^a^Swiss-Prot primary accession number

^b^Mean ± S.D., control refers to untreated cells.

^c^Only peptides with a statistically significant Mascot score (p < 0.05) were considered

The pattern of morphine-responsive proteins was clearly dependent upon the duration of exposure of the cells to morphine. In fact, only 8 proteins were found to be significantly regulated at 2 or 3 time points. Vacuolar ATP synthase subunit B and bifunctional purine biosynthesis protein were upregulated at 6 and 24 h. Rab7 was downregulated at 6 and 24 h while guanine nucleotide binding protein β subunit 2-like 1 (also known as RACK1) was downregulated at 6 and 72 h. Adenylosuccinate synthetase 2 was upregulated at 6 h and downregulated at 72 h. Septin 11 and spot 24 containing a mixture of Rab GDI β and mitochondrial-processing peptidase β were upregulated at 6 h, returned to baseline at 24 h and showed only a minor upregulation at 72 h. Finally, only malate dehydrogenase was downregulated at each time point.

## Discussion

Overall, the present whole cell proteomic analysis has identified 53 proteins from 55 spots whose relative volume is modified upon morphine treatment in SH-SY5Y cells. Once mixtures are excluded, 45 morphine-responsive proteins can be classified according to cellular function (see Additional file 1). These are involved in cell metabolism, organization of the cytoskeleton, vesicle trafficking, transcriptional regulation, protein translation, folding and degradation, and cell signaling.

The pattern of morphine-responsive proteins appears to be dependent on the duration of morphine treatment (compare Tables 1, 2 and 3), indicating that cellular adaptation to chronic morphine is a dynamic process. Such dynamic adaptation has previously been documented in the striatum of chronically morphine-treated rats, wherein various members of the Fos family of transcription factors are sequentially upregulated, implying that distinct sets of genes are regulated over time [3]. Alternatively, it is possible that changes could have been missed at some time point due to technical limitation. For example, RACK1 was found to be downregulated at each time point but this downregulation reached statistical significance only for the 6 and 72 h time points. The difficulty to detect subtle variations in low abundance proteins is a well known limitation of large scale proteomic studies. Thus, the present whole cell analysis failed to detect changes in heterotrimeric G protein β subunits and prohibitin whose relative abundance was previously shown to be decreased in the detergent-resistant membrane (DRM) raft fraction isolated from morphine-treated cells [11]. This is readily explained by the fact that standard sample preparation for 2-DE (the present study), which involves incubation with Triton X-100 at 4°C followed by high speed centrifugation, has eliminated the DRM raft fraction and the proteins which this fraction is enriched in, including heterotrimeric G protein subunits [11]. In addition, the present study has focused onto the ~950 most abundant and soluble proteins of the cell, which represent only 1 to 10% of the total protein species likely to be expressed in a cell. This illustrates the necessity of combining both whole cell and sub-proteome targeted analyses to obtain a clear picture of the proteome of a given cell type.

Four recently published proteomic analyzes have addressed chronic morphine effects in the brain [12-15]. Seven of our morphine-responsive proteins, or closely related isoforms, were also found to be modified in these studies: ATP synthase beta chain, vacuolar ATP synthase subunit B, malate dehydrogenase, triosephosphate isomerase, rab GDP dissociation inhibitor beta, peptidyl-prolyl isomerase, and septin-11. These are indications that at least some of the brain protein changes associated with chronic morphine treatment *in vivo *can be reproduced in our cellular model *in vitro *after a relatively short time of morphine exposure. Conversely, some of the new changes identified in our present *in vitro *study may provide hints as to what neuronal processes may be regulated upon chronic morphine treatment *in vivo*.

Ascribing a precise role for the identified proteins in mediating specific chronic morphine effects in neurons would be too speculative at this stage. However it appears noteworthy to highlight possible links between selected proteins identified in this study and known effects of chronic morphine. Similar to what was recently described for cocaine [16], some of morphine transcriptional effects could be mediated at the level of histone acetylation and chromatin remodeling since two histone acetyltransferase subunits (RuvB-like 1 and histone acetyltransferase type B subunit 2) are upregulated after 24 h of treatment. The variation in 20S proteasome subunit composition observed after 72 h of morphine is consistent with previous studies demonstrating a role for the ubiquitin/proteasome pathway in mediating chronic morphine effects [11,17].

Such transcriptional and post-translational regulations can lead to numerous changes in cellular function among which alterations in cellular architecture [18,19], vesicle trafficking [4] and signal transduction pathways are essential for drug-induced neuronal plasticity. Regarding neuronal architecture and synaptic remodelling, potentially interesting morphine targets are ERM proteins, septins [20] and Rho-GTPase-activating protein 1 [21]. Concerning vesicular transport, the SNAP-alpha downregulation observed in our study is consistent with the recent observation that chronic morphine could induce an inhibition of SNARE complex formation [22]. Morphine could also act at the level of small GTPases of the rab family which are essential for vesicle trafficking. The observed increase in rab GDP dissociation inhibitor beta after 72 h of morphine could result in an alteration of the amount of active rab proteins available for neurotransmitter release [23]. These data are all consistent with chronic morphine modifying neurotransmitter secretion and synapse efficacy.

Finally, chronic morphine induced a change in abundance of several signaling proteins: heterotrimeric G protein subunit α i2, serine-threonine kinase receptor associated protein and receptor for activated C kinase 1 (RACK1). The downregulation of Gα i2 has already been described in our previous study [11] and will not be discussed further. The observed upregulation of serine-threonine kinase receptor associated protein provides a new link between morphine signaling and the phosphatidylinositol 3-kinase/Akt pathway [24]. RACK1 downregulation in brains of morphine-treated rats has been described previously but it has only been discussed in terms of changes in PKC activity since it was its only known function at this time [25]. Since then, RACK1 has been characterized as a scaffolding protein for many proteins involved in cell signaling [26]. Alterations in RACK1 abundance could thus coordinate changes in distinct signaling pathways and its role in molecular adaptations to drugs of abuse deserves further investigation [27].

## Conclusion

In conclusion, our differential proteomics study has identified several candidate proteins that could be implicated in the molecular adaptation of neurons to chronic morphine treatment. In particular, changes in vesicular trafficking and proteasome-dependent protein degradation are known to be critical for various forms of synaptic plasticity such as long term potentiation or depression [28,29]. These adaptations as well as their role in the development of drug dependence have now to be validated in animal models of addiction.

## Methods

### Cell culture and treatment

Human SH-SY5Y neuroblastoma cells were transfected with N-terminal T7-tagged human MOP receptor in pRC-CMV using lipofectamine [11]. Stably transfected G418-resistant cells were grown in high glucose DMEM (Gibco BRL) containing 10% fetal calf serum, 50 μg/ml gentamicine (Gibco BRL), and 400 μg/ml G418 (Gibco BRL), in 5% CO_2 _at 37°C. Cells in 14 cm dishes were treated by diluting morphine sulphate directly in the culture medium. The cell density was adjusted so that the cultures were nearly confluent at the end of each treatment. For 72 h treatment, medium was changed and fresh morphine was added every day. Four independent experiments were performed for each treatment time.

### Sample preparation for 2-DE

Following treatment, the culture medium was removed and the cells were scraped into PBS and centrifuged at 1000 × g for 10 min at 4°C. The supernatant was discarded and the pellet was frozen and stored at -80°C for 2 hours. Cells were then resuspended in 500 μl of lysis buffer [50 mM Tris-HCl pH 7.4, 150 mM NaCl, 10 mM EDTA, proteinase inhibitors (Complete Mini tablets, Roche) and 1% (v:v) Triton X-100 (Sigma)], and protein extraction allowed to proceed for 3 h at 4°C. The extract was centrifuged at 20,000 × g for 20 min at 4°C in order to remove cell debris and insoluble material. The supernatant was delipidated and desalted using methanol/chloroform precipitation by mixing with 2 ml of methanol, 500 μl of chloroform and 1.5 ml of water, and centrifuging at 20,000 × g for 5 min, at room temperature. The pellet was washed with 1.5 ml of methanol and vacuum-dried. Proteins were then resuspended in 400 μl of 2-DE buffer (Cellular and Organelle Membrane Solubilizing Reagent, Sigma), reduced for 1 h at room temperature by adding tributylphosphine to a final concentration of 5 mM, and alkylated in the dark for 1.5 h at room temperature by adding iodoacetamide to a final concentration of 15 mM. Protein concentration was determined by using the Bradford assay (Biorad).

### Two-dimensional gel electrophoresis

The reduced and alkylated protein samples (300 μg) were used to rehydrate 13 cm pH 3–10 NL Immobilized pH Gradient (IPG) strips (Amersham Biosciences). 1.2 μl of IPG buffer pH 3–10 NL (Amersham Biosciences) and 2 μl of 1% (w:v) orange G were added to 250 μl of sample before application to the strip. Strips were focused on the IPGphor IEF (isoelectrofocusing) Cell (Amersham Biosciences) for 80000 Vh. After 6 h of passive rehydration and 6 h of active rehydration (30 V), the voltage was set to 300 V for 4 h. It was then increased from 300 to 8000 V during 8 h, followed by additional hours at 8000 V (until the desired volt × hour product is reached). Focused IPG strips were equilibrated in SDS-equilibration buffer containing 6 M urea, 2% (w:v) SDS, 50 mM Tris-HCl (pH 8.8), 30% (v:v) glycerol and 0.01% (w/v) bromophenol blue as a tracking dye for 2 × 10 min. The equilibrated strips were loaded onto 16 × 16 cm, 1 mm thick, 12% polyacrylamide SDS gels. Runs were performed at 10 mA/gel (SE600 Ruby, Amersham Biosciences) until the bromophenol blue reached the bottom of the gel. Gels were then stained overnight using Coomassie colloidal blue (1 g/l, Sigma) and destained for 1 h in 1% (v/v) acetic acid solution.

### Image analysis

Gels were scanned using a GS-800 Calibrated Densitometer (Biorad) and analyzed using ImageMaster Platinum software (Amersham Biosciences). Spots were detected on quadruplicate gels and manually edited. Normalization was performed by dividing the volume (OD × surface) of each spot by the total volume of every spot on the gel to give a %volume value. Gels were then matched and each group of equivalent spots was compared. In a preliminary experiment, 4 independent control gel were compared. Pair-wise comparison of spot %volumes gave linear regressions of the type y = ax + b where a = 1.03 ± 0.06 and b = -0.0019 ± 0.0034, and a correlation coefficient of 0.95 ± 0.011. Average coefficient of variation (SD/mean × 100) for all spot %volumes was 19.8 ± 10.1. This level of reproducibility is indicative of a statistical power of 80 to 90% for a sample size of 4 and a 0.05 p-value [30]. Having assessed the reliability of our analyses, %volumes of spots from control gels were compared to those of spots from treated gels using a Student unpaired t test. Groups of spots showing a statistically significant difference in relative volume (p < 0.05, df = 6) between control and morphine-treated samples were first visually controlled in order to make sure that %volume changes were not due to spot detection or matching errors. Confirmed spots were then selected for identification by mass spectrometry.

### In-gel protein digestion

Spots of interest were manually excised from a representative gel. The gel pieces were washed with water and destained first in 160 μl of 50% (v:v) acetronitrile (ACN) in water then in 160 μl of 50% (v:v) ACN in 0.1 M NH_4_HCO_3_, dehydrated with 80 μl ACN and dried in a vacuum centrifuge. Gel pieces were rehydrated in a sufficient covering volume of modified trypsin solution (12.5 ng/μl in 12.5 mM NH_4_HCO_3_; Promega) and incubated overnight at 37°C. Prior to peptide extraction, 0.5 μl of the tryptic digest was spotted onto the MALDI target plate. Peptides were extracted two times at 37°C for 30 min with shaking, first using 50% 25 mM NH_4_HCO_3 _/50% ACN and then 5% formic acid/50% ACN in water. The peptide mixture was concentrated in a vacuum centrifuge to a final volume of about 10 μl.

### MALDI-TOF MS analysis

MALDI-TOF MS analyses were performed on a MALDI-TOF/TOF instrument (4700 Proteomics Analyzer; Applied Biosystems, Foster City, CA). 0.5 μl of tryptic digest supernatant was loaded onto the MALDI target plate and air dried. 0.3 μl of matrix solution (α-cyano-4-hydroxycinnamic acid; 5 mg/ml in H_2_O/acetonitrile/TFA, 50:50:0.1) was then added. Mass spectra were acquired in an automated positive reflector mode from *m*/*z *700 to *m*/*z *3500. Trypsin autolytic peptides (*m/z *842.51 and 2211.10) were used to internally calibrate each spectrum to a mass accuracy within 30 ppm. Spectra were analyzed using GPS Explorer (version 3.5, Applied Biosystems) which acts as an interface between the Oracle database containing raw spectra and a local copy of the Mascot search engine (version 2.0 ; Matrix Science, London, U.K.). Peptide peaks with a signal/noise ratio greater than 10 were searched against human sequences in the Swiss-Prot database (Release 48 from September 2005 ; 194 317 entries). No missed cleavage was allowed and the data were searched using carbamidomethylation of cysteine as fixed modification whereas oxidation of methionine and N-acetylation of the proteins were considered as variable modifications. A protein was considered correctly identified if the Protein Score Confidence Interval calculated by the GPS Explorer Results Browser was greater than 99% (see Additional file 2).

### NanoLC-ESI-Q-TOF MS/MS analysis

When MALDI-TOF data were not conclusive (protein score not significant), protein identification was confirmed by MS/MS analyses. Tryptic peptide extracts were subjected to nanoLC-MS/MS analysis on an ESI-Q-TOF mass spectrometer (QSTAR XL, Applied Biosystems) operating in positive mode with a 2.1 kV spray voltage. Chromatographic separation was performed onto a 75 μm ID × 15 cm PepMap C18 column (Dionex/LC Packings, USA) at a flow rate of 200 nL/minute using a linear gradient of increasing ACN in water (5–50%) over 40 min with 0.1% formic acid as ion pairing agent. Data were acquired with Analyst QS (version 1.1, Applied Biosystems). MS spectra were acquired for one second. For each MS spectrum, the two most intense multiple charged peaks were selected for generation of subsequent Collision Induced Dissociation (CID) mass spectra. The collision energy was automatically adjusted based upon peptide charge and mass to charge (*m/z*) ratio. A dynamic exclusion window was applied to prevent repetitive selection of the same ions within 30 sec. Data were analyzed using Analyst QS software (version 1.1) and MS/MS centroid peak lists were generated using the Mascot.dll script (version 1.6b13). MS/MS centroid peaks were thresholded at 0% of the base peak. Data were searched against human sequences in the Swiss-Prot/TrEMBL database as of October 2005 using Mascot (version 2.0). Peptide tolerance in MS and MS/MS modes was 0.5 Da. Trypsin was designated as the protease, and up to two missed cleavages were allowed. Carbamidomethylation of cysteine was searched as fixed modification, while oxidation of methionine and amino-terminal protein acetylation were allowed as variable modifications. Identification was considered positive if the protein was identified on the bases of at least two peptides with a score greater then the significance threshold score determined by the Mascot Search program (>36) (see Additional file 2).

## Competing interests

The author(s) declare that they have no competing interests.

## Authors' contributions

All authors read and approved the final manuscript.

JN carried out the proteomic studies, data analysis, and participated in drafting the manuscript.

SU carried out the mass spectrometric studies and helped to draft the manuscript.

KC participated in experiment and data analysis.

BM supervised mass spectrometric experiments.

JCM participated in the design of the study and in drafting the manuscript.

LM designed the study, supervised experiments and data analysis, and drafted the manuscript.

## Supplementary Material

Additional file 1 Functional classification of proteins regulated by chronic morphine. Click here for file

Additional file 2 Complete mass spectrometry data for identification. Click here for file
