# Supplementary material for: Effect of long-term exposure of SH-SY5Y cells to morphine: a whole cell proteomic analysis
Source: Proteome Sci. 2006 Dec 21;4:23. doi: 10.1186/1477-5956-4-23 (PMC1766345; doi:10.1186/1477-5956-4-23)
Supplement: Additional file 1 — Functional classification of proteins regulated by chronic morphine. [file 1477-5956-4-23-S1.pdf]

Supplementary Table 1. Functional classification of proteins regulated by chronic morphine.

**Metabolic enzymes**	
Glycerol 3-phosphate dehydrogenase	sn-glycerol 3-P + NAD+ = glycerone phosphate + NADH.
Alpha-enolase	Multifunctional enzyme that, as well as its role in glycolysis, plays a part in various processes such as growth control.
Adenylosuccinate synthetase 2	Plays an important role in the de novo pathway of purine nucleotide biosynthesis.
Bifuntional purine biosynthesis protein	Nucleotide biosynthesis; IMP biosynthesis.
Ornithine aminotransferase	L-ornithine + a 2-oxo acid = L-glutamate 5-semialdehyde + an L-amino acid.
Beta-succinyl CoA synthetase	Involved in nucleotide synthesis.
Galactokinase	Major enzyme for galactose metabolism.
Acyl-CoA hydrolase	Catalyzes the hydrolysis of acyl-CoAs to the free fatty acid and coenzyme A (CoASH).
Biliverdin reductase A	Bilirubin + NAD(P)+ = biliverdin + NAD(P)H.
3-mercaptopyruvate sulfurtransferase	Transfer of a sulfur ion to cyanide or to other thiol compounds.
Malate dehydrogenase, cytoplasmic	(S)-malate + NAD+ = oxaloacetate + NADH.
Triosephosphate isomerase	D-glyceraldehyde 3-phosphate = glycerone phosphate.
**Nuclear proteins, transcriptional regulation**	
Lamin A/C	Component of the nuclear lamina. Provides a framework for the nuclear envelope and may interact with chromatin.
RuvB-like 1	Component of the NuA4 histone acetyltransferase complex involved in transcriptional activation of select genes.
Histone acetyltransferase type B subunit 2	Core-histone-binding subunit that target histone acetyltransferases to their histone substrates.
Proliferation-associated protein 2G4	Involved in transcriptional repression, inhibits proliferation.
Nuclear protein Hcc-1	May participate in important transcriptional or translational control of cell growth, metabolism and carcinogenesis.
**Protein translation, folding and degradation**	
Elongation factor G 1	Promotes the GTP-dependent translocation of the nascent protein chain from the A-site to the P-site of the ribosome.
Elongation factor 1-gamma	Probably plays a role in anchoring the elongation complex to other cellular components.
ATP-dependent RNA helicase DDX1	Regulation of translational initiation.
Heat shock cognate 71 kDa protein	Chaperone
T-complex protein 1, zeta subunit	Molecular chaperone; assists the folding of proteins upon ATP hydrolysis.
40 kDa peptidyl-prolyl cis-trans isomerase	PPIases accelerate the folding of proteins.
Endoplasmic reticulum protein ERp29	Plays an important role in the processing of secretory proteins within the endoplasmic reticulum.
Ubiquitin-like 1 activating enzyme E1A	The dimeric enzyme acts as a UBL1 (SUMO) E1 ligase.
Proteasome subunit alpha type 3	Part of a multicatalytic proteinase complex involved in the degradation of poly-ubiquitynated proteins.
Proteasome subunit beta type 6	See proteasome subunit alpha type 3

Supplementary Table 1. (continued)

**Cytoskeleton-associated proteins**	
Ezrin	Probably involved in connections of major cytoskeletal structures to the plasma membrane.
Radixin	Probably plays a crucial role in the binding of the barbed end of actin filaments to the plasma membrane.
Dihydropyrimidinase-related protein 3	Binds to F-actin, potentially regulates the cytoskeleton.
Septin-11	Involved in cytokinesis and in a variety of other processes involving organization of the cell cortex and exocytosis.
Septin-2	See septin-11
Rho-GTPase-activating protein 1	GTPase activator for the Rho, Rac and Cdc42 proteins, converting them to the putatively inactive GDP-bound state.
**Vesicle-associated proteins**	
Vacuolar ATP synthase subunit B, brain isoform	V-ATPase is responsible for acidifying a variety of intracellular compartments in eukaryotic cells.
ATP synthase beta chain	Produces ATP from ADP in the presence of a proton gradient across the membrane. Catalytic subunit.
Rab GDP dissociation inhibitor beta	Regulates the GDP/GTP exchange reaction of most Rab proteins by inhibiting the dissociation of GDP from them.
Synaptic vesicle membrane protein VAT-1 homolog	Homologous to VAT-1, the major protein of the synaptic vesicles of the electric organ of Torpedo californica.
Alpha-soluble NSF attachment protein	SNAP-alpha, Required for vesicular transport between the endoplasmic reticulum and the Golgi apparatus.
Ras-related protein Rab-7	Involved in late endocytic transport. Contributes to the maturation of phagosomes (acidification).
**Cell signaling**	
Guanine nucleotide binding protein, alpha inhibiting activity polypeptide 2	Alpha i2 subunit of heterotrimeric G proteins
Guanine nucleotide binding protein beta subunit 2-like 1	RACK1, anchors the activated PKC to the cytoskeleton. Involved in the organisation of signaling complexes.
Serine-threonine kinase receptor associated protein	Role in phosphatidylinositol 3-kinase/PDK1 and TGF-beta signaling pathways.
**Micellaneous**	
Arsenical pump-driving ATPase	Anion-transporting ATPase. Catalyzes the extrusion of arsenite.
Annexin A5	Anticoagulant protein that acts as an indirect inhibitor of the thromboplastin-specific complex.
Platelet-activating factor acetylhydrolase IB gamma subunit	Inactivates paf by removing the acetyl group at the sn-2 position (important for brain development).

Description of protein function is based on the annotations from the Swiss-Prot database.
