# Supplementary material for: Effect of long-term exposure of SH-SY5Y cells to morphine: a whole cell proteomic analysis
Source: Proteome Sci. 2006 Dec 21;4:23. doi: 10.1186/1477-5956-4-23 (PMC1766345; doi:10.1186/1477-5956-4-23)
Supplement: Additional file 2 — Complete mass spectrometry data for identification. [file 1477-5956-4-23-S2.pdf]

SUPPLEMENTARY TABLES Complete MS data for the 55 spots

Spot #1. MALDI MS data - 32% coverage

**Gel Idx/Pos**	129/F9	**Instr./Gel Origin**	gloin/Spotset040318	**Process Status**	Analysis Succeeded
**Plate [#] Name**	[1] Sandrine	**Instrument Sample Name**		**Spectra**	1

Rank	Protein Name	Accession No.	Protein Score	Protein Score C. I. %	Total Ion C. I. %	Protein MW	Protein PI	Pep. Count	Intensity Matched
1	(Q92499) ATP-dependent RNA helicase DDX1 (EC 3.6.1.-) (DEAD box protein 1) (DEAD box protein retino	DDX1_HUMAN	205	100		83349.1	6.81	25	8.787

Peptide Information

Calc. Mass	Obsrv. Mass	± da	± ppm	Start Seq.	End Seq.	Sequence	Modification
749.3828	749.3836	0.0008	1	312	317	YIDNPK	
770.325	770.3269	0.0019	2	637	642	GCYNTR	Carbamidomethyl (C)[2]
785.3788	785.3816	0.0028	4	466	472	TDDVHAK	
786.4508	786.4327	-0.0181	-23	282	288	FLPNAPK	
816.4111	816.416	0.0049	6	597	602	QNYVHR	
829.489	829.4874	-0.0016	-2	359	365	LNLSQVR	
836.4512	836.4356	-0.0156	-19	253	260	DGFVALSK	
844.3519	844.3633	0.0114	14	118	123	EWHGCR	Carbamidomethyl (C)[5]
862.4668	862.4539	-0.0129	-15	261	268	APDGYIVK	
884.52	884.5104	-0.0096	-11	289	296	ALIVEPSR	
947.5043	947.4942	-0.0101	-11	350	358	LDDLVSTGK	
974.473	974.4672	-0.0058	-6	163	172	FGFGFGGTGK	
1005.4887	1005.4891	0.0004	0	679	687	VPVDEFDGK	
1109.4833	1109.515	0.0317	29	91	98	WQMNPYDR	
1111.6833	1111.6782	-0.0051	-5	320	330	ELLIIGGVAAR	
1125.4783	1125.4958	0.0175	16	91	98	WQMNPYDR	Oxidation (M)[3]
1146.5101	1146.5295	0.0194	17	240	248	FNFGEEEFK	
1153.5493	1153.5643	0.015	13	506	514	MDQAIIFCR	Carbamidomethyl (C)[8]
1165.6034	1165.6029	-0.0005	0	568	577	FLICTDVAAR	Carbamidomethyl (C)[4]
1169.5442	1169.55	0.0058	5	506	514	MDQAIIFCR	Carbamidomethyl (C)[8], Oxidation (M)[1]
1260.6769	1260.6731	-0.0038	-3	224	234	NQALFPACVLK	Carbamidomethyl (C)[8]

1272.6794	1272.6616	-0.0178	-14	297	307	ELAEQTLNNIK	
1385.6768	1385.6791	0.0023	2	269	281	SQHSGNAQVTQTK	
1572.6794	1572.6848	0.0054	3	537	549	GHQFSCVCLHGDR	Carbamidomethyl (C)[6,8]
1685.7369	1685.7452	0.0083	5	99	114	GSAFAIGSDGLCCQSR	Carbamidomethyl (C)[12,13]
1823.7588	1823.7845	0.0257	14	133	146	HYYEVSCHDQGLCR	Carbamidomethyl (C)[7,13]
2025.0399	2025.0309	-0.009	-4	433	451	GEDSVPDTVHHVVVPVNPK	

Spot #2. MALDI MS data - 16% coverage

**Gel Idx/Pos**	34/B15	**Instr./Gel Origin**	gloin/Spotset050705	**Process Status**	Analysis Succeeded
**Plate [#] Name**	[1] Sandrine	**Instrument Sample Name**		**Spectra**	1

Rank	Protein Name	Accession No.	Protein Score	Protein Score C. I. %	Total Ion C. I. %	Protein MW	Protein PI	Pep. Count	Intensity Matched
1	(Q96RP9) Elongation factor G 1, mitochondrial precursor (mEF-G 1) (Elongation factor G1)	EFG1_HUMAN	87	99.997		84193.6	6.58	13	5.732

Peptide Information

Calc. Mass	Obsrv. Mass	± da	± ppm	Start Seq.	End Seq.	Sequence	Modification
741.3638	741.3672	0.0034	5	244	250	AAATDHR	
746.3977	746.4053	0.0076	10	186	191	ALQQMR	
820.4345	820.4318	-0.0027	-3	341	347	ILMNSSR	
939.453	939.4606	0.0076	8	381	388	GDTIYNTR	
952.4482	952.4543	0.0061	6	542	551	QSGGAGQYGK	
983.5308	983.5253	-0.0055	-6	365	372	FGQLTYVR	
1043.5731	1043.5725	-0.0006	-1	213	221	GIVDLIEER	
1047.5469	1047.5452	-0.0017	-2	235	243	YGEIPAELR	
1135.4724	1135.4691	-0.0033	-3	711	719	GEYTMEYSR	
1218.6729	1218.6409	-0.032	-26	735	745	YLEATGQLPVK	
1331.6742	1331.6611	-0.0131	-10	348	359	DNSHPFVGLAFK	
1500.7482	1500.739	-0.0092	-6	222	234	AIYFDGDFGQIVR	
1602.8163	1602.7937	-0.0226	-14	527	540	ETITAPVPFDFTHK	

Spot #3. MALDI MS data - 15% coverage

**Gel Idx/Pos**	132/F15	**Instr./Gel Origin**	gloin/Spotset041117	**Process Status**	Analysis Succeeded
**Plate [#] Name**	[1] Sandrine	**Instrument Sample Name**		**Spectra**	1

Rank	Protein Name	Accession No.	Protein Score	Protein Score C. I. %	Total Ion C. I. %	Protein MW	Protein PI	Pep. Count	Intensity Matched
1	(P15311) Ezrin (p81) (Cytovillin) (Villin-2)	EZRI_HUMAN	80	99.987		69338.7	5.95	10	8.48

Peptide Information

Calc. Mass	Obsrv. Mass	± da	± ppm	Start Seq.	End Seq.	Sequence	Modification
848.3533	848.3588	0.0055	6	165	170	DQWEDR	
914.5305	914.5333	0.0028	3	427	434	IALLEEAR	
924.4308	924.4307	-0.0001	0	350	356	LQDYEEK	
976.5461	976.5391	-0.007	-7	27	34	QLFDQVVK	
1002.5214	1002.5143	-0.0071	-7	363	370	ELSEQIQR	
1104.5836	1104.5773	-0.0063	-6	237	245	IGFPWSEIR	
1175.6068	1175.6056	-0.0012	-1	171	179	IQVWHAEHR	
1182.5942	1182.5905	-0.0037	-3	263	272	APDFVFYAPR	
1509.6863	1509.6674	-0.0189	-13	547	558	THNDIIHNENMR	Oxidation (M)[11]
1651.8174	1651.7863	-0.0311	-19	412	426	SQEQLAAELAEYTAK	

Spot #4. ESI MS/MS data

RADI_HUMAN **Mass: 68635 Score: 440 Queries matched: 16**
(P35241) Radixin

Check to include this hit in error tolerant search or archive report

Query		Observed	Mr(exp	Mr(calc)	Delta	Miss	Score	Expect	Rank	Peptide
	21	412,24	822,5	822,51	-0,04	1	23	0,79	2	PKPINVR
	25	427,7	853,4	853,43	-0,04	0	19	1,6	1	GYSTWLK
	30	438,22	874,4	874,45	-0,04	0	27	0,46	1	ENPLQFK
	31	443,75	885,5	885,52	-0,02	0	21	1,7	1	IALLEEAK
	36	480,28	958,5	958,59	-0,04	1	39	0,027	1	FVIKPIDK
	37	487,22	972,4	972,49	-0,05	0	33	0,1	1	ALELDQER
	38	488,76	975,5	975,54	-0,04	0	35	0,071	1	QLFDQVVK
	39	494,75	987,5	987,52	-0,03	0	42	0,015	1	QIEEQTIK
	40	502,26	1003	1002,55	-0,04	1	25	0,63	1	KENPLQFK
	42	561,26	1121	1120,54	-0,04	0	53	0,001	1	AFAAQEDLEK
	43	577,74	1153	1153,51	-0,04	1	46	0,0048	1	QRIDEFEAM + Oxidation (M)
	46	416,84	1248	1247,58	-0,07	0	18	2,8	1	IQNWHEEHR
	47	471,55	1412	1411,72	-0,08	1	11	12	1	EIHKPGYLANDR
	48	496,9	1488	1487,78	-0,09	2	15	5,3	2	RKPDTIEVQQMK + Oxidation (M)
	49	499,25	1495	1494,78	-0,07	1	34	0,069	1	KTQNDVLHAENVK
	52	885,42	1769	1769,02	-0,2	2	8	23	7	VKKQLQALSSELAQAR

Spot #5. MALDI MS data - 32% coverage

**Gel Idx/Pos**	133/F17	**Instr./Gel Origin**	gloin/Spotset041117	**Process Status**	Analysis Succeeded
**Plate [#] Name**	[1] Sandrine	**Instrument Sample Name**		**Spectra**	1

Rank	Protein Name	Accession No.	Protein Score	Protein Score C. I. %	Total Ion C. I. %	Protein MW	Protein PI	Pep. Count	Intensity Matched
1	(P02545) Lamin A/C (70 kDa lamin)	LAMA_HUMAN	205	100		74379.8	6.57	18	15.572

Peptide Information

Calc. Mass	Obsrv. Mass	± da	± ppm	Start Seq.	End Seq.	Sequence	Modification
849.4828	849.4869	0.0041	5	42	48	LAVYIDR	
919.438	919.4316	-0.0064	-7	428	435	SSFSQHAR	
974.4788	974.4797	0.0009	1	379	386	LLEGEEER	
1023.5105	1023.5065	-0.004	-4	209	216	NIYSEELR	
1028.5735	1028.563	-0.0105	-10	241	249	LADALQELR	
1043.548	1043.5431	-0.0049	-5	124	133	EGDLIAAQAR	
1089.5535	1089.5679	0.0144	13	51	60	SLETENAGLR	
1165.5483	1165.5398	-0.0085	-7	79	89	AAYEAELGDAR	
1182.6113	1182.6002	-0.0111	-9	157	166	TLEGELHDLR	
1291.6277	1291.6337	0.006	5	472	482	QNGDDPLLTYR	
1347.6725	1347.661	-0.0115	-9	367	377	LALDMEIHAYR	Oxidation (M)[5]
1359.6863	1359.6857	-0.0006	0	12	25	SGAQASSTPLSPTR	
1363.6172	1363.6179	0.0007	1	516	527	AQNTWGCGNSLR	Carbamidomethyl (C)[7]
1507.7421	1507.7256	-0.0165	-11	528	541	TALINSTGEEVAMR	Oxidation (M)[13]
1566.7506	1566.74	-0.0106	-7	628	644	SVGGSGGGSFGDNLVTR	
1752.8623	1752.8468	-0.0155	-9	281	296	NSNLVGAAHEELQQSR	
1909.9211	1909.9113	-0.0098	-5	352	366	MQQQLDEYQELLDIK	Oxidation (M)[1]
2365.1589	2365.1538	-0.0051	-2	598	624	ASASGSGAQVGGPISSGSSASSVT VTR	

Spot #6. no ID

Spot #7. MALDI MS data - 22% coverage

**Gel Idx/Pos**	123/E22	**Instr./Gel Origin**	gloin/Spotset040318	**Process Status**	Analysis Succeeded
**Plate [#] Name**	[1] Sandrine	**Instrument Sample Name**		**Spectra**	1

Rank	Protein Name	Accession No.	Protein Score	Protein Score C. I. %	Total Ion C. I. %	Protein MW	Protein PI	Pep. Count	Intensity Matched
1	(P43304) Glycerol-3-phosphate dehydrogenase, mitochondrial precursor (EC 1.1.99.5) (GPD-M) (GPDH-M)	GPDM_HUMAN	108	100		81276.7	6.98	17	5.513

Peptide Information

Calc. Mass	Obsrv. Mass	± da	± ppm	Start Seq.	End Seq.	Sequence	Modification
751.4573	751.4573	0	0	114	120	LIHGGVR	
754.3842	754.3895	0.0053	7	139	144	EALHER	
795.4722	795.4583	-0.0139	-17	716	722	VPIPVDR	
817.46	817.4473	-0.0127	-16	573	579	IVELMGR	
833.4549	833.4478	-0.0071	-9	573	579	IVELMGR	Oxidation (M)[5]
848.4108	848.4159	0.0051	6	609	615	SEQLTDR	
936.4785	936.4799	0.0014	1	129	135	LDIEQYR	
947.4428	947.4496	0.0068	7	707	714	TAEENLDR	
974.4901	974.4837	-0.0064	-7	589	596	QEQLETAR	
1147.647	1147.6443	-0.0027	-2	641	650	GFITIVDVQR	
1229.6346	1229.6067	-0.0279	-23	200	209	ALEHFPMLQK	Oxidation (M)[7]
1254.5784	1254.583	0.0046	4	381	390	NYLSCDVEVR	Carbamidomethyl (C)[5]
1278.6324	1278.6324	0	0	272	282	DVLTGQEFDVR	
1344.7006	1344.7014	0.0008	1	616	627	SEISLLPSDIDR	
1352.6879	1352.6954	0.0075	6	179	190	LYDLVAGSNCLK	Carbamidomethyl (C)[10]
1415.6294	1415.6305	0.0011	1	543	554	EYACTAVDMISR	Carbamidomethyl (C)[4]
1431.6243	1431.637	0.0127	9	543	554	EYACTAVDMISR	Carbamidomethyl (C)[4], Oxidation (M)[9]
1536.7474	1536.7531	0.0057	4	285	298	CVINATGPFTDSVR	Carbamidomethyl (C)[1]
1641.8959	1641.8734	-0.0225	-14	558	572	LAFLNVQAAEEALPR	

Spot #8. MALDI MS data - 23% coverage

**Gel Idx/Pos**	148/F24	**Instr./Gel Origin**	gloin/Spotset041117	**Process Status**	Analysis Succeeded
**Plate [#] Name**	[1] Sandrine	**Instrument Sample Name**		**Spectra**	1

Rank	Protein Name	Accession No.	Protein Score	Protein Score C. I. %	Total Ion C. I. %	Protein MW	Protein PI	Pep. Count	Intensity Matched
1	(P11142) Heat shock cognate 71 kDa protein (Heat shock 70 kDa protein 8)	HSP7C_HUMAN	127	100		71082.3	5.37	12	12.728

Peptide Information

Calc. Mass	Obsrv. Mass	± da	± ppm	Start Seq.	End Seq.	Sequence	Modification
1081.5676	1081.5603	-0.0073	-7	349	357	LLQDFFNGK	
1199.6742	1199.6715	-0.0027	-2	160	171	DAGTIAGLNVLR	
1228.6281	1228.6351	0.007	6	26	36	VEIIANDQGNR	
1251.619	1251.6118	-0.0072	-6	237	246	MVNHFIAEFK	Oxidation (M)[1]
1253.6161	1253.6122	-0.0039	-3	302	311	FEELNADLFR	
1319.5936	1319.599	0.0054	4	540	550	NSLESYAFNMK	Oxidation (M)[10]
1481.807	1481.786	-0.021	-14	329	342	SQIHDIVLVGGSTR	
1487.7013	1487.705	0.0037	2	37	49	TTPSYVAFTDTER	
1632.7826	1632.7789	-0.0037	-2	113	126	SFYPEEVSSMVLTK	Oxidation (M)[10]
1649.7952	1649.785	-0.0102	-6	57	71	NQVAMNPTNTVFDAK	
1665.79	1665.7844	-0.0056	-3	57	71	NQVAMNPTNTVFDAK	Oxidation (M)[5]
1691.7256	1691.7255	-0.0001	0	221	236	STAGDTHLGGEDFDNR	
1981.9978	1981.9989	0.0011	1	138	155	TVTNAVVTVPAYFNDSQR	

Spot #9. MALDI MS data - 30% coverage

**Gel Idx/Pos**	134/F19	**Instr./Gel Origin**	gloin/Spotset041117	**Process Status**	Analysis Succeeded
**Plate [#] Name**	[1] Sandrine	**Instrument Sample Name**		**Spectra**	1

Rank	Protein Name	Accession No.	Protein Score	Protein Score C. I. %	Total Ion C. I. %	Protein MW	Protein PI	Pep. Count	Intensity Matched
1	(P31939) Bifunctional purine biosynthesis protein PURH [Includes: Phosphoribosylaminoimidazolecarbo	PUR9_HUMAN	163	100		65088.5	6.27	15	9.017

Peptide Information

Calc. Mass	Obsrv. Mass	± da	± ppm	Start Seq.	End Seq.	Sequence	Modification
890.5094	890.5076	-0.0018	-2	91	97	LDFNLIR	
892.4886	892.4904	0.0018	2	15	22	TGLVEFAR	
903.4716	903.4767	0.0051	6	200	207	GVSQMPLR	Oxidation (M)[5]
1008.5049	1008.5216	0.0167	17	462	468	ANYWWLR	
1092.5619	1092.5566	-0.0053	-5	469	477	HHPQVLSMK	Oxidation (M)[8]
1163.642	1163.6425	0.0005	0	295	305	TLTPISAAYAR	
1245.5529	1245.5387	-0.0142	-11	80	90	NIPEDNADMAR	
1309.6973	1309.6923	-0.005	-4	98	108	VVACNLYPFVK	Carbamidomethyl (C)[4]
1332.7158	1332.6932	-0.0226	-17	2	14	APGQLALFSVSDK	
1355.7906	1355.7876	-0.003	-2	67	79	TLHPAVHAGILAR	
1384.7291	1384.7252	-0.0039	-3	438	451	NGQVIGIGAGQQSR	
1607.7733	1607.8048	0.0315	20	50	64	DVSELTGFPEMLGGR	
1623.7683	1623.7679	-0.0004	0	50	64	DVSELTGFPEMLGGR	Oxidation (M)[11]
1847.9497	1847.9615	0.0118	6	267	285	HVSPAGAAVGIPLSEDEAK	
2034.9192	2034.9255	0.0063	3	178	194	AFTHTAQYDEAISDYFR	
2103.0327	2102.9929	-0.0398	-19	208	225	YGMNPHQTPAQLYTLQPK	Oxidation (M)[3]

Spot #10. MALDI MS data - 37% coverage

**Gel Idx/Pos**	16/A4	**Instr./Gel Origin**	gloin/Spotset050705	**Process Status**	Analysis Succeeded
**Plate [#] Name**	[1] Sandrine	**Instrument Sample Name**		**Spectra**	1

Rank	Protein Name	Accession No.	Protein Score	Protein Score C. I. %	Total Ion C. I. %	Protein MW	Protein PI	Pep. Count	Intensity Matched
1	(P02545) Lamin-A/C (70 kDa lamin)	LMNA_HUMAN	210	100		74379.8	6.57	25	17.779

Peptide Information

Calc. Mass	Obsrv. Mass	± da	± ppm	Start Seq.	End Seq.	Sequence	Modification
849.4828	849.485	0.0022	3	42	48	LAVYIDR	
887.4832	887.4734	-0.0098	-11	226	233	LVEIDNGK	
906.3986	906.3932	-0.0054	-6	1	7	METPSQR	N-Acetyl (Protein)[0], Oxidation (M)[1]
908.4359	908.458	0.0221	24	202	208	EELDFQK	
918.4526	918.4504	-0.0022	-2	322	329	DLEDSLAR	
919.438	919.4384	0.0004	0	428	435	SSFSQHAR	
972.5109	972.5122	0.0013	1	389	397	LSPSPTSQR	
974.4788	974.489	0.0102	10	379	386	LLEGEEER	
1023.5105	1023.5074	-0.0031	-3	209	216	NIYSEELR	
1028.5735	1028.5693	-0.0042	-4	241	249	LADALQELR	
1032.5143	1032.5111	-0.0032	-3	182	189	QLQDEMLR	
1043.548	1043.5525	0.0045	4	124	133	EGDLIAAQAR	
1048.5092	1048.5028	-0.0064	-6	182	189	QLQDEMLR	Oxidation (M)[6]
1089.5535	1089.5508	-0.0027	-2	51	60	SLETENAGLR	
1131.5277	1131.5245	-0.0032	-3	33	41	EDLQELNDR	
1148.5793	1148.5707	-0.0086	-7	63	72	ITESEEVVSR	
1165.5483	1165.5475	-0.0008	-1	79	89	AAYEAELGDAR	
1182.6113	1182.6069	-0.0044	-4	157	166	TLEGELHDLR	
1291.6277	1291.6248	-0.0029	-2	472	482	QNGDDPLLTYR	
1331.6776	1331.6703	-0.0073	-5	367	377	LALDMEIHAYR	
1347.6726	1347.6595	-0.0131	-10	367	377	LALDMEIHAYR	Oxidation (M)[5]
1359.6863	1359.6846	-0.0017	-1	12	25	SGAQASSTPLSPTR	
1363.6172	1363.6157	-0.0015	-1	516	527	AQNTWGCGNSLR	Carbamidomethyl (C)[7]

1374.6284	1374.6185	-0.0099	-7	250	260	AQHEDQVEQYK	
1491.7472	1491.7395	-0.0077	-5	528	541	TALINSTGEEVAMR	
1536.6747	1536.6754	0.0007	0	458	470	SNEDQSMGNWQIK	
1552.6697	1552.6608	-0.0089	-6	458	470	SNEDQSMGNWQIK	Oxidation (M)[7]
1752.8623	1752.8586	-0.0037	-2	281	296	NSNLVGAAHEELQQSR	

Spot #11. no ID

Spot #12. MALDI MS data - 29% coverage

**Gel Idx/Pos**	128/F7	**Instr./Gel Origin**	gloin/Spotset041117	**Process Status**	Analysis Succeeded
**Plate [#] Name**	[1] Sandrine	**Instrument Sample Name**		**Spectra**	1

Rank	Protein Name	Accession No.	Protein Score	Protein Score C. I. %	Total Ion C. I. %	Protein MW	Protein PI	Pep. Count	Intensity Matched
1	(P40227) T-complex protein 1, zeta subunit (TCP-1-zeta) (CCT-zeta) (CCT-zeta-1) (Tcp20) (HTR3) (Acu	TCPZ_HUMAN	119	100		58312.7	6.25	16	9.448

Peptide Information

Calc. Mass	Obsrv. Mass	± da	± ppm	Start Seq.	End Seq.	Sequence	Modification
757.4566	757.4586	0.002	3	307	313	EGIVALR	
784.4239	784.4222	-0.0017	-2	359	364	FTFIEK	
800.4624	800.4646	0.0022	3	28	34	GLQDVLR	
840.4938	840.4918	-0.002	-2	381	387	HTLTQIK	
904.525	904.5239	-0.0011	-1	287	294	GFVVINQK	
916.5098	916.509	-0.0008	-1	145	152	ETLIDVAR	
937.5213	937.5184	-0.0029	-3	208	216	GLVLDHGAR	
1006.5237	1006.5153	-0.0084	-8	45	54	MLVSGAGDIK	Oxidation (M)[1]
1021.516	1021.515	-0.001	-1	199	207	SETDTSLIR	
1076.5986	1076.5945	-0.0041	-4	129	137	ALQFLEEVK	
1078.5779	1078.5811	0.0032	3	117	126	IITEGFEAAK	
1191.568	1191.5651	-0.0029	-2	241	250	TEVNSGFFYK	
1262.6627	1262.6538	-0.0089	-7	295	306	GIDPFSLDALSK	
1498.7649	1498.7599	-0.005	-3	104	116	QADLYISEGLHPR	
1761.9381	1761.9385	0.0004	0	449	464	VLAQNSGFDLQETLVK	
2045.9736	2045.983	0.0094	5	224	240	VEDAYILTCNVSLEYEK	Carbamidomethyl (C)[9]

Spot #13. MALDI MS data - 40% coverage

**Gel Idx/Pos**	97/D18	**Instr./Gel Origin**	gloin/Spotset041117	**Process Status**	Analysis Succeeded
**Plate [#] Name**	[1] Sandrine	**Instrument Sample Name**		**Spectra**	1

Rank	Protein Name	Accession No.	Protein Score	Protein Score C. I. %	Total Ion C. I. %	Protein MW	Protein PI	Pep. Count	Intensity Matched
1	(Q14195) Dihydropyrimidinase-related protein 3 (DRP-3) (Unc-33-like phosphoprotein) (ULIP protein)	DPYL3_HUMAN	160	100		62323.2	6.04	17	11.882

Peptide Information

Calc. Mass	Obsrv. Mass	± da	± ppm	Start Seq.	End Seq.	Sequence	Modification
766.4569	766.4555	-0.0014	-2	556	563	IVAPPGGR	
773.39	773.3878	-0.0022	-3	512	520	GGTPAGSAR	
922.5145	922.5153	0.0008	1	391	397	IFNLYPR	
1009.5247	1009.5192	-0.0055	-5	488	496	MADLHAVPR	
1025.5197	1025.5134	-0.0063	-6	488	496	MADLHAVPR	Oxidation (M)[1]
1031.548	1031.541	-0.007	-7	259	268	SAADLISQAR	
1141.6398	1141.6263	-0.0135	-12	441	451	GAPLVVICQGK	Carbamidomethyl (C)[8]
1309.7474	1309.7273	-0.0201	-15	44	56	QIGDNLIVPGGVK	
1446.657	1446.6691	0.0121	8	82	94	GMTTVDDFFQGTK	
1462.6519	1462.6455	-0.0064	-4	82	94	GMTTVDDFFQGTK	Oxidation (M)[2]
1657.7778	1657.7775	-0.0003	0	497	511	GMYDGPVFDLTTTPK	Oxidation (M)[2]
1710.8591	1710.839	-0.0201	-12	452	467	IMLEDGNLHVTQGAGR	
1726.8541	1726.8486	-0.0055	-3	452	467	IMLEDGNLHVTQGAGR	Oxidation (M)[2]
1741.8062	1741.8048	-0.0014	-1	375	390	MDENQFVAVTSTNAAK	Oxidation (M)[1]
1748.8086	1748.8091	0.0005	0	346	361	DNFTAIPEGTNGVEER	
1779.9309	1779.8872	-0.0437	-25	239	254	AITIASQTNCPLYVTK	Carbamidomethyl (C)[10]
1915.9647	1915.9633	-0.0014	-1	401	418	ISVGSDSDLVIWDPDAVK	
2008.9181	2008.9226	0.0045	2	424	440	NHQSAAEYNIFEGMELR	
2024.9131	2024.9115	-0.0016	-1	424	440	NHQSAAEYNIFEGMELR	Oxidation (M)[14]
2030.989	2030.9908	0.0018	1	532	550	NLHQSGFSLSGTQVDEGVR	
2365.0903	2365.0957	0.0054	2	24	43	IVNDDQSFYADIYMEDGLIK	Oxidation (M)[14]

Spot #14. MALDI MS data - 54% coverage

**Gel Idx/Pos**	118/E12	**Instr./Gel Origin**	gloin/Spotset040318	**Process Status**	Analysis Succeeded
**Plate [#] Name**	[1] Sandrine	**Instrument Sample Name**		**Spectra**	1

Rank	Protein Name	Accession No.	Protein Score	Protein Score C. I. %	Total Ion C. I. %	Protein MW	Protein PI	Pep. Count	Intensity Matched
1	(P21281) Vacuolar ATP synthase subunit B, brain isoform (EC 3.6.3.14) (V-ATPase B2 subunit) (Vacuol	VATB2_HUMAN	199	100		56807	5.57	19	13.611

Peptide Information

Calc. Mass	Obsrv. Mass	± da	± ppm	Start Seq.	End Seq.	Sequence	Modification
873.4788	873.4677	-0.0111	-13	30	37	EQALAVSR	
877.4526	877.4529	0.0003	0	38	44	NYLSQPR	
937.4407	937.4406	-0.0001	0	404	412	SAIGEGMTR	Oxidation (M)[7]
1104.5354	1104.5326	-0.0028	-3	121	130	TPVSEDMLGR	
1120.5303	1120.5272	-0.0031	-3	121	130	TPVSEDMLGR	Oxidation (M)[7]
1298.6045	1298.6038	-0.0007	-1	110	120	TSCEFTGDILR	Carbamidomethyl (C)[3]
1308.6331	1308.6349	0.0018	1	461	471	NFIAQGPYENR	
1437.7373	1437.7383	0.001	1	495	506	IPQSTLSEFYPR	
1520.7954	1520.7881	-0.0073	-5	94	108	AVVQVFEGTSGIDAK	
1556.8319	1556.825	-0.0069	-4	68	81	YAEIVHLTLPDGTK	
1596.9108	1596.9093	-0.0015	-1	387	400	QIYPPINVLPSLSR	
1647.9427	1647.9376	-0.0051	-3	49	64	TVSGVNGPLVILDHVK	
1757.8778	1757.8701	-0.0077	-4	277	291	LALTTAEFLAYQCEK	Carbamidomethyl (C)[13]
1912.8785	1912.8755	-0.003	-2	322	337	GFPGYMYTDLATIYER	Oxidation (M)[6]
1924.8857	1924.8917	0.006	3	414	430	DHADVSNQLYACYAIGK	Carbamidomethyl (C)[12]
1934.0051	1934.0121	0.007	4	292	308	HVLVILTDMSSYAEALR	Oxidation (M)[9]
2000.0923	2000.0896	-0.0027	-1	8	29	GIVNGAAPELPVPTGGPAVGAR	
2178.1487	2178.1494	0.0007	0	189	208	IPIFSAAGLPHNEIAAQICR	Carbamidomethyl (C)[19]
2353.2173	2353.2173	0	0	437	457	AVVGEEALTSDDLLYLEFLQK	
2441.1687	2441.1377	-0.031	-13	164	185	IYPEEMIQTGISAIDGMNSIAR	Oxidation (M)[6,17]

Spot #15. ESI MS/MS data

RHG01_HUMAN **Mass: 50461** **Score: 322** **Queries matched: 10**

(Q07960) Rho-GTPase-activating protein 1 (GTPase-activating protein rhoOGAP) (Rho-related small GTP

Check to include this hit in error tolerant search or archive report

Query	Observed	Mr(expt)	Mr(calc)	Delta	Miss	Score	Expect	Rank	Peptide
124	353,74	705,46	705,44	0,02	0	26	0,5	1	LLGYLK
160	366,23	730,44	730,42	0,02	0	46	0,0078	1	DAAITLK
210	388,22	774,42	774,41	0,01	0	29	0,37	2	LASIDEK
237	400,7	799,39	799,38	0,02	0	32	0,14	1	YDDFLK
379	463,29	924,57	924,54	0,03	0	41	0,014	1	LEQLGIPR
398	483,28	964,54	964,52	0,02	0	27	0,47	1	IIVFSACR
443	559,83	1117,65	1117,61	0,04	0	37	0,043	1	AINPINTFTK
461	399,89	1196,66	1196,64	0,02	0	24	0,79	1	SSSPELVTHLK
464	605,82	1209,62	1209,6	0,02	0	47	0,0041	1	HQIVEVAGDDK
475	431,55	1291,61	1291,6	0,02	0	13	11	1	MPPSHQLDHSK + Oxidation (M)

Spot #16. MALDI MS data - 66% coverage

**Gel Idx/Pos**	159/G21	**Instr./Gel Origin**	gloin/Spotset040318	**Process Status**	Analysis Succeeded
**Plate [#] Name**	[1] Sandrine	**Instrument Sample Name**		**Spectra**	1

Rank	Protein Name	Accession No.	Protein Score	Protein Score C. I. %	Total Ion C. I. %	Protein MW	Protein PI	Pep. Count	Intensity Matched
1	(Q9Y265) RuvB-like 1 (EC 3.6.1.-) (49-kDa TATA box-binding protein-interacting protein) (49 kDa TBP	RUVB1_HUMAN	238	100		50538.4	6.02	21	24.287

Peptide Information

Calc. Mass	Obsrv. Mass	± da	± ppm	Start Seq.	End Seq.	Sequence	Modification
878.4842	878.4831	-0.0011	-1	15	22	IASHSHVK	
930.489	930.4849	-0.0041	-4	446	453	ILADQQDK	
1059.568	1059.564	-0.004	-4	23	33	GLGLDESGLAK	
1080.6411	1080.6532	0.0121	11	65	76	AVLLAGPPGTGK	
1138.5562	1138.5548	-0.0014	-1	109	117	TEVLMENFR	
1154.551	1154.5457	-0.0053	-5	109	117	TEVLMENFR	Oxidation (M)[5]
1230.6761	1230.6733	-0.0028	-2	47	57	EACGVIVELIK	Carbamidomethyl (C)[3]
1241.5905	1241.5842	-0.0063	-5	363	372	TMLYTPQEMK	
1257.5854	1257.5873	0.0019	2	363	372	TMLYTPQEMK	Oxidation (M)[2]
1273.5803	1273.5796	-0.0007	-1	363	372	TMLYTPQEMK	Oxidation (M)[2,9]
1276.6783	1276.6727	-0.0056	-4	172	182	LDPSIFESLQK	
1300.6604	1300.6622	0.0018	1	34	46	QAASGLVGQENAR	
1385.7998	1385.7931	-0.0067	-5	77	90	TALALAIAQELGSK	
1530.889	1530.8875	-0.0015	-1	405	418	YSVQLLTPANLLAK	
1687.9377	1687.9414	0.0037	2	318	333	ALESSIAPIVIFASNR	
1708.8064	1708.8049	-0.0015	-1	428	441	EHVEEISELFYDAK	
1734.8907	1734.8867	-0.004	-2	185	201	VEAGDVIYIEANSGAVK	
1765.8281	1765.824	-0.0041	-2	250	265	PQGGQDILSMMGQLMK	Oxidation (M)[10,11]
1921.9978	1921.9991	0.0013	1	233	249	EIIQDVTLHDLDVANAR	
1942.9288	1942.937	0.0082	4	91	107	VPFCPMVGSEVYSTEIK	Carbamidomethyl (C)[4]
1948.9974	1948.9976	0.0002	0	340	357	GTEDITSPHGIPLDLLDR	
1958.9238	1958.9301	0.0063	3	91	107	VPFCPMVGSEVYSTEIK	Carbamidomethyl (C)[4], Oxidation (M)[6]
2324.1729	2324.1694	-0.0035	-2	379	400	AQTEGINISEEALNHLGEIGTK	

2390.0745	2390.0764	0.0019	1	206	225	CDTYATEFDLEAEEYVPLPK	Carbamidomethyl (C)[1]
2689.1643	2689.165	0.0007	0	129	152	EVYEGEVTELTPCETENPMGGYGK	Carbamidomethyl (C)[13]
2705.1592	2705.157	-0.0022	-1	129	152	EVYEGEVTELTPCETENPMGGYGK	Carbamidomethyl (C)[13], Oxidation (M)[19]

Spot #17. MALDI MS data - 37% coverage

**Gel Idx/Pos**	98/D20	**Instr./Gel Origin**	gloin/Spotset041117	**Process Status**	Analysis Succeeded
**Plate [#] Name**	[1] Sandrine	**Instrument Sample Name**		**Spectra**	1

Rank	Protein Name	Accession No.	Protein Score	Protein Score C. I. %	Total Ion C. I. %	Protein MW	Protein PI	Pep. Count	Intensity Matched
1	(Q9NVA2) Septin-11	SEP11_HUMAN	122	100		49521.3	6.38	15	14.361

Peptide Information

Calc. Mass	Obsrv. Mass	± da	± ppm	Start Seq.	End Seq.	Sequence	Modification
771.3744	771.3705	-0.0039	-5	286	291	EQTHTR	
811.3953	811.401	0.0057	7	342	347	QMFVMR	
827.3902	827.3824	-0.0078	-9	342	347	QMFVMR	Oxidation (M)[2]
869.4073	869.4037	-0.0036	-4	301	307	LEEMGFK	Oxidation (M)[4]
876.4243	876.4172	-0.0071	-8	279	285	VNMEDLR	
880.4312	880.4305	-0.0007	-1	292	297	HYELYR	
892.4193	892.4231	0.0038	4	279	285	VNMEDLR	Oxidation (M)[3]
980.6502	980.6505	0.0003	0	175	183	VNIIPIIAK	
1077.5575	1077.5609	0.0034	3	327	335	NEFLGELQK	
1152.5432	1152.5421	-0.0011	-1	137	145	SLFNYHDTR	
1224.5854	1224.5814	-0.004	-3	83	92	SYELQESNVR	
1286.6296	1286.6163	-0.0133	-10	54	64	STLMDTLFNTK	Oxidation (M)[4]
1378.6484	1378.6432	-0.0052	-4	386	396	ELEEEVNNFQK	
1555.7135	1555.7107	-0.0028	-2	65	78	FESDPATHNEPGVR	
1619.8639	1619.8551	-0.0088	-5	95	109	LTIVDTVGFGDQINK	
1957.0098	1956.9971	-0.0127	-6	399	417	AAAQLLQSQAQQSGAQQTK	
2140.1033	2140.0925	-0.0108	-5	14	33	NLSLSGHVGFDSLPDQLVNK	

Spot #18. MALDI MS data - 48% coverage

**Gel Idx/Pos**	143/F14	**Instr./Gel Origin**	gloin/Spotset041117	**Process Status**	Analysis Succeeded
**Plate [#] Name**	[1] Sandrine	**Instrument Sample Name**		**Spectra**	1

Rank	Protein Name	Accession No.	Protein Score	Protein Score C. I. %	Total Ion C. I. %	Protein MW	Protein PI	Pep. Count	Intensity Matched
1	(P06576) ATP synthase beta chain, mitochondrial precursor (EC 3.6.3.14)	ATPB_HUMAN	188	100		56524.6	5.26	18	34.498

Peptide Information

Calc. Mass	Obsrv. Mass	± da	± ppm	Start Seq.	End Seq.	Sequence	Modification
779.4232	779.4234	0.0002	0	1	7	MLGFVGR	
975.5621	975.5507	-0.0114	-12	202	212	IGLFGGAGVGK	
1038.5942	1038.5878	-0.0064	-6	134	143	IPVGPETLGR	
1088.635	1088.6215	-0.0135	-12	189	198	VVDLLAPYAK	
1262.6409	1262.6335	-0.0074	-6	110	121	TIAMDGTEGLVR	
1278.6359	1278.6243	-0.0116	-9	110	121	TIAMDGTEGLVR	Oxidation (M)[4]
1385.7094	1385.6968	-0.0126	-9	144	155	IMNVIGEPIDER	
1401.7042	1401.694	-0.0102	-7	144	155	IMNVIGEPIDER	Oxidation (M)[2]
1406.681	1406.6716	-0.0094	-7	226	239	AHGGYSVFAGVGER	
1435.7539	1435.7479	-0.006	-4	311	324	FTQAGSEVSALLGR	
1439.7893	1439.7838	-0.0055	-4	282	294	VALTGLTVAEYFR	
1457.8396	1457.8224	-0.0172	-12	213	225	TVLIMELINNVAK	
1473.8345	1473.8257	-0.0088	-6	213	225	TVLIMELINNVAK	Oxidation (M)[5]
1601.8104	1601.8049	-0.0055	-3	265	279	VALVYGQMNEPPGAR	
1617.8053	1617.8022	-0.0031	-2	265	279	VALVYGQMNEPPGAR	Oxidation (M)[8]
1650.9174	1650.9092	-0.0082	-5	95	109	LVLEVAQHLGESTVR	
1815.8694	1815.86	-0.0094	-5	407	422	IMDPNIVGSEHYDVAR	
1831.8643	1831.8605	-0.0038	-2	407	422	IMDPNIVGSEHYDVAR	Oxidation (M)[2]
1921.9655	1921.9565	-0.009	-5	295	310	DQEGQDVLLFIDNIFR	
1988.0334	1988.0281	-0.0053	-3	388	406	AIAELGIYPAVDPLDSTSR	
2023.0106	2023.017	0.0064	3	463	480	FLSQPFQVAEVFTGHMGK	
2039.0055	2039.0038	-0.0017	-1	463	480	FLSQPFQVAEVFTGHMGK	Oxidation (M)[16]
2076.9905	2076.9875	-0.003	-1	242	259	EGNDLYHEMIESGVINLK	Oxidation (M)[9]

2266.0842	2266.0747	-0.0095	-4	325	345	IPSAVGYQPTLATDMGTMQER	
2282.0791	2282.0798	0.0007	0	325	345	IPSAVGYQPTLATDMGTMQER	Oxidation (M)[15]
2298.074	2298.0894	0.0154	7	325	345	IPSAVGYQPTLATDMGTMQER	Oxidation (M)[15,18]

Spot #19. MALDI MS data - 32 & 25% coverage

**Gel Idx/Pos**	115/E6	**Instr./Gel Origin**	gloin/Spotset040318	**Process Status**	Analysis Succeeded
**Plate [#] Name**	[1] Sandrine	**Instrument Sample Name**		**Spectra**	1

Rank	Protein Name	Accession No.	Protein Score	Protein Score C. I. %	Total Ion C. I. %	Protein MW	Protein PI	Pep. Count	Intensity Matched
1	(P49189) 4-trimethylaminobutyraldehyde dehydrogenase (EC 1.2.1.47) (TMABADH) (Aldehyde dehydrogenas	AL9A1_HUMAN	117	100		54679.3	5.69	15	12.294

Peptide Information

Calc. Mass	Obsrv. Mass	± da	± ppm	Start Seq.	End Seq.	Sequence	Modification
785.4879	785.4864	-0.0015	-2	76	82	ILLEAAR	
804.3345	804.3515	0.017	21	369	374	DGYYMR	
820.3293	820.3377	0.0084	10	369	374	DGYYMR	Oxidation (M)[5]
848.426	848.4266	0.0006	1	31	38	AFEPATGR	
851.4509	851.4473	-0.0036	-4	304	310	FTEEVVK	
1019.4761	1019.4736	-0.0025	-2	375	382	PCVLTNCR	Carbamidomethyl (C)[2,7]
1085.595	1085.5876	-0.0074	-7	50	59	EVNLAVQNAK	
1103.5215	1103.5239	0.0024	2	20	30	VEPADASGTEK	
1128.5895	1128.5862	-0.0033	-3	317	326	IGDPLLEDTR	
1212.5929	1212.5941	0.0012	1	39	49	VIATFTCSGEK	Carbamidomethyl (C)[7]
1226.6085	1226.6053	-0.0032	-3	228	239	VSFTGSVPTGMK	Oxidation (M)[11]
1243.6569	1243.6552	-0.0017	-1	472	481	VTIEYYSQLK	
1540.7754	1540.7673	-0.0081	-5	412	426	ANDTTFGLAAGVFTR	
1561.7567	1561.7513	-0.0054	-3	353	366	VLCGGDIYVPEDPK	Carbamidomethyl (C)[3]
1610.8173	1610.8195	0.0022	1	2	15	STGTFVVSQPLNYR	N-Acetyl (Protein)[0]
1839.8616	1839.8608	-0.0008	0	259	274	SPLIIFSDCDMNNAVK	Carbamidomethyl (C)[9], Oxidation (M)[11]

Rank	Protein Name	Accession No.	Protein Score	Protein Score C. I. %	Total Ion C. I. %	Protein MW	Protein PI	Pep. Count	Intensity Matched
2	(Q9GZL7) WD-repeat protein 12 (YTM1 homolog)	WDR12_HUMAN	88	99.998		48190.9	5.57	12	5.339

Peptide Information

Calc. Mass	Obsrv. Mass	± da	± ppm	Start Seq.	End Seq.	Sequence	Modification
758.4155	758.4122	-0.0033	-4	2	7	AQLQTR	N-Acetyl (Protein)[0]
806.4002	806.3906	-0.0096	-12	312	319	LASGSTDR	

816.3603	816.3645	0.0042	5	181	186	ALHCCR	Carbamidomethyl (C)[4,5]
832.4562	832.4457	-0.0105	-13	125	131	IWSLEGK	
863.4403	863.4468	0.0065	8	1	7	MAQLQTR	Oxidation (M)[1]
917.505	917.4888	-0.0162	-18	240	247	TEQLGLTR	
986.4036	986.4048	0.0012	1	204	211	FCSGSWDK	Carbamidomethyl (C)[2]
1019.4792	1019.4736	-0.0056	-5	414	423	YSPTTSHVGA	
1119.5681	1119.5634	-0.0047	-4	282	291	VWDVESGSLK	
1342.6637	1342.6576	-0.0061	-5	377	388	APLYDLAAHEDK	
1468.6954	1468.6897	-0.0057	-4	109	121	GAEEWILTGSYDK	
1557.7504	1557.7545	0.0041	3	187	203	GHAGSVDSIAVDGSGTK	

Spot #20. MALDI MS data - 60% coverage

**Gel Idx/Pos**	126/F3	**Instr./Gel Origin**	gloin/Spotset040318	**Process Status**	Analysis Succeeded
**Plate [#] Name**	[1] Sandrine	**Instrument Sample Name**		**Spectra**	1

Rank	Protein Name	Accession No.	Protein Score	Protein Score C. I. %	Total Ion C. I. %	Protein MW	Protein PI	Pep. Count	Intensity Matched
1	(P06733) Alpha-enolase (EC 4.2.1.11) (2-phospho-D-glycerate hydro-lyase) (Non-neural enolase) (NNE)	ENOA_HUMAN	232	100		47350.4	6.99	21	21.887

Peptide Information

Calc. Mass	Obsrv. Mass	± da	± ppm	Start Seq.	End Seq.	Sequence	Modification
766.3729	766.3658	-0.0071	-9	9	14	EIFDSR	
773.3424	773.3426	0.0002	0	262	268	SPDDPSR	
800.3824	800.3759	-0.0065	-8	256	261	YDLDFK	
806.4518	806.4489	-0.0029	-4	406	411	YNQLLR	
810.4468	810.4364	-0.0104	-13	64	70	AVEHINK	
899.556	899.5488	-0.0072	-8	71	79	TIAPALVSK	
904.4622	904.4579	-0.0043	-5	412	419	IEEELGSK	
1007.5012	1007.5017	0.0005	0	335	342	SCNCLLLK	Carbamidomethyl (C)[2,4]
1143.6156	1143.6128	-0.0028	-2	183	192	IGAEVYHNLK	
1280.5861	1280.5874	0.0013	1	92	102	LMIEMDGTENK	
1296.5811	1296.5946	0.0135	10	92	102	LMIEMDGTENK	Oxidation (M)[2]
1312.5759	1312.5853	0.0094	7	92	102	LMIEMDGTENK	Oxidation (M)[2,5]
1406.7162	1406.7153	-0.0009	-1	15	27	GNPTVEVDLFTSK	
1425.7261	1425.7238	-0.0023	-2	269	280	YISPDQLADLYK	
1519.8301	1519.8116	-0.0185	-12	105	119	FGANAILGVSLAVCK	Carbamidomethyl (C)[14]
1540.7828	1540.7838	0.001	1	239	252	VVIGMDVAASEFFR	
1556.7777	1556.7775	-0.0002	0	239	252	VVIGMDVAASEFFR	Oxidation (M)[5]
1633.8214	1633.8197	-0.0017	-1	343	357	VNQIGSVTESLQACK	Carbamidomethyl (C)[14]
1804.9438	1804.9481	0.0043	2	32	49	AAVPSGASTGIYEALELR	
1939.9768	1939.9802	0.0034	2	162	178	LAMQEFMILPVGAANFR	Oxidation (M)[3,7]
1960.9246	1960.9285	0.0039	2	202	220	DATNVGDEGGFAPNILENK	
2033.0549	2033.0597	0.0048	2	306	325	FTASAGIQVVGDDLTVTNPK	

2353.1592	2353.1541	-0.0051	-2	372	393	SGETEDTFIADLVVGLCTGQIK	Carbamidomethyl (C)[17]
2510.1147	2510.1345	0.0198	8	285	305	DYPVVSIEDPFDQDDWGAWQK	

Spot #21. ESI MS/MS data

ENOA_HUMAN **Mass: 47350** **Score: 515** **Queries matched: 14**
(P06733) Alpha-enolase (EC 4.2.1.11) (2-phospho-D-glycerate hydro-lyase) (Non-neural enolase) (NNE)

Check to include this hit in error tolerant search or archive report

Query	Observed	Mr(expt)	Mr(calc)	Delta	Miss	Score	Expect	Rank	Peptide
22	352,69	703,36	703,4	-0,04	0	30	0,25	1	GVPLYR
33	383,66	765,31	765,37	-0,05	0	32	0,11	1	EIFDSR
39	400,68	799,34	799,38	-0,04	0	44	0,0096	1	YDLDFK
40	401,22	800,42	800,46	-0,04	0	47	0,0051	1	EGLELLK
42	403,71	805,4	805,44	-0,04	0	15	7,7	2	YNQLLR
65	450,25	898,49	898,55	-0,06	0	26	0,54	1	TIAPALVSK
66	452,71	903,4	903,45	-0,05	0	48	0,0036	1	IEEELGSK
73	480,73	959,44	959,49	-0,05	0	32	0,16	1	LNVTEQEK
83	504,22	1006,43	1006,49	-0,06	0	31	0,15	1	SCNCLLLK
104	381,85	1142,54	1142,61	-0,07	0	27	0,48	1	IGAEVYHNLK
125	703,82	1405,63	1405,71	-0,08	0	48	0,003	1	GNPTVEVDLFTSK
127	713,32	1424,62	1424,72	-0,1	0	59	0,00025	1	YISPDQLADLYK
133	545,25	1632,74	1632,81	-0,08	0	42	0,011	1	VNQIGSVTESLQACK
136	602,28	1803,81	1803,94	-0,13	0	35	0,058	1	AAVPSGASTGIYEALELR

Spot #22. MALDI MS data - 30% coverage

**Gel Idx/Pos**	133/F17	**Instr./Gel Origin**	gloin/Spotset040318	**Process Status**	Analysis Succeeded
**Plate [#] Name**	[1] Sandrine	**Instrument Sample Name**		**Spectra**	1

Rank	Protein Name	Accession No.	Protein Score	Protein Score C. I. %	Total Ion C. I. %	Protein MW	Protein PI	Pep. Count	Intensity Matched
1	(Q16576) Histone acetyltransferase type B subunit 2 (Retinoblastoma binding protein P46) (Retinobla	RBBP7_HUMAN	91	99.999		48132.3	4.89	11	7.781

Peptide Information

Calc. Mass	Obsrv. Mass	± da	± ppm	Start Seq.	End Seq.	Sequence	Modification
791.3967	791.3809	-0.0158	-20	114	119	IECEIK	Carbamidomethyl (C)[3]
894.4567	894.4461	-0.0106	-12	15	21	VINEEYK	
950.4764	950.4777	0.0013	1	251	257	LMIWDTR	Oxidation (M)[2]
998.5054	998.4849	-0.0205	-21	309	316	LHTFESHK	
1067.5228	1067.5237	0.0009	1	120	128	INHEGEVNR	
1142.5476	1142.5472	-0.0004	0	102	113	GEFGGFGSVTGK	
1284.5413	1284.5453	0.004	3	5	14	EMFEDTVEER	
1300.5361	1300.5371	0.001	1	5	14	EMFEDTVEER	Oxidation (M)[2]
1428.7303	1428.7211	-0.0092	-6	131	142	YMPQNPHIIATK	Oxidation (M)[2]
1471.7314	1471.7336	0.0022	1	143	155	TPSSDVLVFDYTK	
2170.9094	2170.9138	0.0044	2	83	101	VHIPNDDAQFDASHCDSDK	Carbamidomethyl (C)[15]
2847.343	2847.3484	0.0054	2	349	375	IGEEQSAEDAEDGPPELLFIHGGHTAK	

Spot #23. MALDI MS data - 34% coverage

**Gel Idx/Pos**	100/D24	**Instr./Gel Origin**	gloin/Spotset041117	**Process Status**	Analysis Succeeded
**Plate [#] Name**	[1] Sandrine	**Instrument Sample Name**		**Spectra**	1

Rank	Protein Name	Accession No.	Protein Score	Protein Score C. I. %	Total Ion C. I. %	Protein MW	Protein PI	Pep. Count	Intensity Matched
1	(P50395) Rab GDP dissociation inhibitor beta (Rab GDI beta) (Guanosine diphosphate dissociation inh	GDIB_HUMAN	111	100		51087.1	6.11	12	4.756

Peptide Information

Calc. Mass	Obsrv. Mass	± da	± ppm	Start Seq.	End Seq.	Sequence	Modification
786.3926	786.3885	-0.0041	-5	30	35	VLHMDR	Oxidation (M)[4]
938.4941	938.4947	0.0006	1	211	218	LYSESLAR	
1029.5251	1029.5046	-0.0205	-20	104	112	VTEGSFVYK	
1125.5973	1125.5739	-0.0234	-21	90	98	MLLYTEVTR	
1141.5922	1141.5717	-0.0205	-18	90	98	MLLYTEVTR	Oxidation (M)[1]
1143.5463	1143.5597	0.0134	12	58	68	IPGSPPESMGR	Oxidation (M)[9]
1344.6833	1344.6772	-0.0061	-5	143	153	FLVYVANFDEK	
1365.7008	1365.696	-0.0048	-4	391	402	DLGTESQIFISR	
1902.8175	1902.8162	-0.0013	-1	194	208	TDDYLDQPCYETINR	Carbamidomethyl (C)[9]
1917.8171	1917.8109	-0.0062	-3	403	418	TYDATTHFETTCDDIK	Carbamidomethyl (C)[12]
2111.0049	2111.0339	0.029	14	330	348	SDIYVCMISFAHNVAAQGK	Carbamidomethyl (C)[6]
2117.0073	2117.0063	-0.001	0	36	54	NPYYGGESASITPLEDLYK	
2199.0571	2199.0557	-0.0014	-1	310	328	NTNDANSCQIIIPQNQVNR	Carbamidomethyl (C)[8]

Spot #24. MALDI MS data - 36% & 25% coverage

Gel Idx/Pos 17/A6 **Instr./Gel Origin** gloin/Spotset050705 **Process Status** Analysis Succeeded
Plate [#] Name [1] Sandrine **Instrument Sample Name** **Spectra** 1

Rank	Protein Name	Accession No.	Protein Score	Protein Score C. I. %	Total Ion C. I. %	Protein MW	Protein PI	Pep. Count	Intensity Matched
1	(P50395) Rab GDP dissociation inhibitor beta (Rab GDI beta) (Guanosine diphosphate dissociation inh	GDIB_HUMAN	126	100		51087.1	6.11	15	11.355

Peptide Information

Calc. Mass	Obsrv. Mass	± da	± ppm	Start Seq.	End Seq.	Sequence	Modification
761.3787	761.3766	-0.0021	-3	270	276	SEGEIAR	
770.3977	770.3915	-0.0062	-8	30	35	VLHMDR	
786.3926	786.3823	-0.0103	-13	30	35	VLHMDR	Oxidation (M)[4]
906.4567	906.4498	-0.0069	-8	157	164	TFEGIDPK	
938.4941	938.4901	-0.004	-4	211	218	LYSESLAR	
1029.5251	1029.5127	-0.0124	-12	104	112	VTEGSFVYK	
1125.5973	1125.5896	-0.0077	-7	90	98	MLLYTEVTR	
1127.5514	1127.5632	0.0118	10	58	68	IPGSPPESMGR	
1136.6133	1136.5957	-0.0176	-15	80	89	FLMANGQLVK	Oxidation (M)[3]
1141.5922	1141.5774	-0.0148	-13	90	98	MLLYTEVTR	Oxidation (M)[1]
1143.5463	1143.5614	0.0151	13	58	68	IPGSPPESMGR	Oxidation (M)[9]
1179.6918	1179.6737	-0.0181	-15	300	309	VICILSHPIK	Carbamidomethyl (C)[3]
1222.6136	1222.6036	-0.01	-8	279	288	QLICDPSYVK	Carbamidomethyl (C)[4]
1344.6833	1344.6815	-0.0018	-1	143	153	FLVYVANFDEK	
1365.7008	1365.6953	-0.0055	-4	391	402	DLGTESQIFISR	
1902.8175	1902.8187	0.0012	1	194	208	TDDYLDQPCYETINR	Carbamidomethyl (C)[9]
1917.8171	1917.8206	0.0035	2	403	418	TYDATTHFETTCDDIK	Carbamidomethyl (C)[12]
2199.0571	2199.0637	0.0066	3	310	328	NTNDANSCQIIIPQNQVNR	Carbamidomethyl (C)[8]

2	(O75439) Mitochondrial-processing peptidase beta subunit, mitochondrial precursor (EC 3.4.24.64) (B	MPPB_HUMAN	94	100	55072.8	6.38	13	8.274

Peptide Information

Calc. Mass	Obsrv. Mass	± da	± ppm	Start Seq.	End Seq.	Sequence	Modification
747.4035	747.3931	-0.0104	-14	473	478	QLPDFK	
809.4151	809.4104	-0.0047	-6	279	285	FTGSEIR	
966.4284	966.4376	0.0092	10	482	488	SNMCWLR	Carbamidomethyl (C)[4]
984.439	984.4647	0.0257	26	425	431	QMLCYNR	Carbamidomethyl (C)[4]
1000.4338	1000.4261	-0.0077	-8	425	431	QMLCYNR	Carbamidomethyl (C)[4], Oxidation (M)[2]
1001.4938	1001.4675	-0.0263	-26	137	144	EQTVYYAK	
1023.6196	1023.6069	-0.0127	-12	462	472	SPAIAAVGPIK	
1034.5299	1034.5261	-0.0038	-4	60	68	VTCLESGLR	Carbamidomethyl (C)[3]
1037.599	1037.5927	-0.0063	-6	433	441	IPIPELEAR	
1085.6201	1085.611	-0.0091	-8	210	219	TILGPTENIK	
1101.5898	1101.585	-0.0048	-4	442	451	IDAVNAETIR	
1348.6102	1348.6095	-0.0007	-1	258	268	FHFGDSLCTHK	Carbamidomethyl (C)[8]
1351.6522	1351.646	-0.0062	-5	388	399	LCTSVTESEVAR	Carbamidomethyl (C)[2]
1713.913	1713.9047	-0.0083	-5	44	59	STQAATQVVLNVPETR	

Spot #25. MALDI MS data - 44% coverage

Gel Idx/Pos	101/E1	Instr./Gel Origin	gloin/Spotset041117	Process Status	Analysis Succeeded
Plate [#] Name	[1] Sandrine	Instrument Sample Name		Spectra	1

Rank	Protein Name	Accession No.	Protein Score	Protein Score C. I. %	Total Ion C. I. %	Protein MW	Protein PI	Pep. Count	Intensity Matched
1	(Q9UQ80) Proliferation-associated protein 2G4 (Cell cycle protein p38-2G4 homolog) (hG4-1)	PA2G4_HUMAN	135	100		44101.3	6.13	15	9.648

Peptide Information

Calc. Mass	Obsrv. Mass	± da	± ppm	Start Seq.	End Seq.	Sequence	Modification
849.3882	849.386	-0.0022	-3	23	30	MGGDIANR	Oxidation (M)[1]
925.4737	925.4733	-0.0004	0	192	199	QHVIDGEK	
932.5159	932.5132	-0.0027	-3	356	364	ALLQSSASR	
981.4887	981.4863	-0.0024	-2	94	101	SDQDYILK	
984.4785	984.479	0.0005	1	264	271	AFFSEVER	
1054.5204	1054.5211	0.0007	1	312	320	EGEFVAQFK	
1097.5448	1097.5581	0.0133	12	273	281	FDAMPFTLR	
1113.5398	1113.5339	-0.0059	-5	273	281	FDAMPFTLR	Oxidation (M)[4]
1182.6047	1182.6053	0.0006	1	145	155	AAHLCAEAALR	Carbamidomethyl (C)[5]
1285.6747	1285.6693	-0.0054	-4	200	210	TIIQNPTDQQK	
1366.689	1366.6812	-0.0078	-6	333	344	ITSGPFEPDLYK	
1391.7174	1391.7089	-0.0085	-6	321	332	FTVLLMPNGPMR	Oxidation (M)[6]
1407.7123	1407.7032	-0.0091	-6	321	332	FTVLLMPNGPMR	Oxidation (M)[6,11]
1629.8635	1629.8572	-0.0063	-4	299	311	HELLQPFNVLYEK	
1851.9368	1851.9186	-0.0182	-10	34	51	SLVEASSSGVSVLSLCEK	Carbamidomethyl (C)[16]
2161.0142	2161.0232	0.009	4	2	20	SGEDEQQEQTIAEDLVVTK	N-Acetyl (Protein)[0]
2264.1079	2264.0964	-0.0115	-5	216	236	AEFEVHEVYAVDVLVSSGEGK	

Spot #26. MALDI MS data - 36% coverage

**Gel Idx/Pos**	102/E3	**Instr./Gel Origin**	gloin/Spotset041117	**Process Status**	Analysis Succeeded
**Plate [#] Name**	[1] Sandrine	**Instrument Sample Name**		**Spectra**	1

Rank	Protein Name	Accession No.	Protein Score	Protein Score C. I. %	Total Ion C. I. %	Protein MW	Protein PI	Pep. Count	Intensity Matched
1	(P26641) Elongation factor 1-gamma (EF-1-gamma) (eEF-1B gamma)	EF1G_HUMAN	140	100		50298.2	6.27	16	29.748

Peptide Information

Calc. Mass	Obsrv. Mass	± da	± ppm	Start Seq.	End Seq.	Sequence	Modification
762.4144	762.4142	-0.0002	0	45	50	TPEFLR	
810.3814	810.3895	0.0081	10	212	218	MAQFDAK	
820.4199	820.4173	-0.0026	-3	220	226	FAETQPK	
821.4515	821.4517	0.0002	0	149	155	TFLVGER	
826.3763	826.3764	0.0001	0	212	218	MAQFDAK	Oxidation (M)[1]
924.4937	924.4858	-0.0079	-9	277	284	DPFAHLPK	
947.4693	947.4684	-0.0009	-1	181	188	QAFPNTNR	
975.5258	975.5247	-0.0011	-1	173	180	QVLEPSFR	
1085.5514	1085.5297	-0.0217	-20	285	293	STFVLDEFK	
1118.682	1118.6742	-0.0078	-7	137	146	ILGLLDAYLK	
1347.7379	1347.735	-0.0029	-2	17	29	ALIAAQYSGAQVR	
1444.7278	1444.7313	0.0035	2	401	413	LDPGSEETQTLVR	
1461.6433	1461.6437	0.0004	0	316	326	DGWSLWYSEYR	
1583.7489	1583.7493	0.0004	0	1	13	AAGTLYTYPENWR	N-Acetyl (Protein)[0]
1609.7944	1609.7971	0.0027	2	189	200	WFLTCINQPQFR	Carbamidomethyl (C)[5]
1684.7754	1684.7733	-0.0021	-1	414	427	EYFSWEGAFQHVGK	
1707.8713	1707.8711	-0.0002	0	30	44	VLSAPPHFHFGQTNR	

Spot #27. MALDI MS data - 31% coverage

**Gel Idx/Pos**	36/B19	**Instr./Gel Origin**	gloin/Spotset050705	**Process Status**	Analysis Succeeded
**Plate [#] Name**	[1] Sandrine	**Instrument Sample Name**		**Spectra**	1

Rank	Protein Name	Accession No.	Protein Score	Protein Score C. I. %	Total Ion C. I. %	Protein MW	Protein PI	Pep. Count	Intensity Matched
1	(Q99536) Synaptic vesicle membrane protein VAT-1 homolog (EC 1.-.-.-)	VAT1_HUMAN	109	100		42121.5	5.88	10	9.179

Peptide Information

Calc. Mass	Obsrv. Mass	± da	± ppm	Start Seq.	End Seq.	Sequence	Modification
731.4232	731.4357	0.0125	17	134	139	VMVLNR	
747.4181	747.4131	-0.005	-7	134	139	VMVLNR	Oxidation (M)[2]
751.3733	751.3743	0.001	1	97	102	QGLYDR	
788.4446	788.4371	-0.0075	-10	297	303	NLMALAR	
804.4396	804.4275	-0.0121	-15	297	303	NLMALAR	Oxidation (M)[3]
951.5873	951.5811	-0.0062	-7	383	391	VLLVPGPEK	
1329.6508	1329.6564	0.0056	4	50	61	CLVLTGFGGYDK	Carbamidomethyl (C)[1]
1338.6293	1338.6318	0.0025	2	85	96	ACGLNFADLMAR	Carbamidomethyl (C)[2]
1354.6243	1354.6174	-0.0069	-5	85	96	ACGLNFADLMAR	Carbamidomethyl (C)[2], Oxidation (M)[10]
1442.8114	1442.8202	0.0088	6	68	82	PAAPPAPGPGQLTLR	
1510.7748	1510.7722	-0.0026	-2	211	225	TVENVTVFGTASASK	
1590.7679	1590.775	0.0071	4	256	271	GVDIVMDPLGGSDTAK	Oxidation (M)[6]
2557.2642	2557.3137	0.0495	19	23	49	TEAASDPQHPAASEGAAAAAASPPLLR	

Spot #28. ESI MS/MS data

PURA2_HUMAN Mass: 50465 Score: 349 Queries matched: 11
(P30520) Adenylosuccinate synthetase 2 (EC 6.3.4.4) (IMP--aspartate ligase 2) (AdSS 2) (AMPSase 2)

Check to include this hit in error tolerant search or archive report

Query	Observed	Mr(expt)	Mr(calc)	Delta	Miss	Score	Expect	Rank	Peptide
7	318,67	635,33	635,36	-0,03	0	34	0,081	1	VGVAYK
9	330,19	658,36	658,38	-0,02	0	20	4,2	1	WIGVGK
38	358,7	715,38	715,42	-0,04	0	30	0,26	1	LIISDR
43	380,22	758,42	758,45	-0,02	1	20	3,1	1	IKPMVR + Oxidation (M)
46	383,18	764,35	764,41	-0,05	0	40	0,02	1	VEVQYK
63	418,21	834,4	834,46	-0,06	0	34	0,085	1	VLANQYK
88	433,7	865,39	865,43	-0,04	0	36	0,044	1	EFGVTTGR
104	442,19	882,37	882,42	-0,04	0	9	23	1	ESMIQLF + Oxidation (M)
110	454,23	906,45	906,48	-0,03	0	42	0,015	1	GIGPVYSSK
137	518,27	1034,53	1034,58	-0,05	1	37	0,042	1	KGIGPVYSSK
167	651,81	1301,61	1301,67	-0,06	0	47	0,0043	1	ELPVNAQNYVR

Spot #29. MALDI MS data - 24% & 19% coverage

**Gel Idx/Pos**	26/A24	**Instr./Gel Origin**	gloin/Spotset050705	**Process Status**	Analysis Succeeded
**Plate [#] Name**	[1] Sandrine	**Instrument Sample Name**		**Spectra**	1

Rank	Protein Name	Accession No.	Protein Score	Protein Score C. I. %	Total Ion C. I. %	Protein MW	Protein PI	Pep. Count	Intensity Matched
1	(P26641) Elongation factor 1-gamma (EF-1-gamma) (eEF-1B gamma)	EF1G_HUMAN	86	99.996		50298.2	6.27	11	3.35

Peptide Information

Calc. Mass	Obsrv. Mass	± da	± ppm	Start Seq.	End Seq.	Sequence	Modification
761.3787	761.3784	-0.0003	0	125	131	QATENAK	
762.4144	762.4112	-0.0032	-4	45	50	TPEFLR	
810.3814	810.3912	0.0098	12	212	218	MAQFDAK	
821.4515	821.4508	-0.0007	-1	149	155	TFLVGER	
947.4693	947.4669	-0.0024	-3	181	188	QAFPNTNR	
975.5258	975.5242	-0.0016	-2	173	180	QVLEPSFR	
1085.5514	1085.5233	-0.0281	-26	285	293	STFVLDEFK	
1347.7379	1347.7308	-0.0071	-5	17	29	ALIAAQYSGAQVR	
1444.7278	1444.7157	-0.0121	-8	401	413	LDPGSEETQTLVR	
1583.7489	1583.734	-0.0149	-9	1	13	AAGTLYTYPENWR	N-Acetyl (Protein)[0]
1707.8713	1707.845	-0.0263	-15	30	44	VLSAPPHFHFGQTNR	

Rank	Protein Name	Accession No.	Protein Score	Protein Score C. I. %	Total Ion C. I. %	Protein MW	Protein PI	Pep. Count	Intensity Matched
2	(Q96Q11) tRNA-nucleotidyltransferase 1, mitochondrial precursor (EC 2.7.7.25) (Mitochondrial tRNA n	TRNT1_HUMAN	64	99.396		50340.2	8.14	10	4.068

Peptide Information

Calc. Mass	Obsrv. Mass	± da	± ppm	Start Seq.	End Seq.	Sequence	Modification
**755.4158**	**755.4126**	**-0.0032**	**-4**	**108**	**114**	**HGTITAR**	
**789.3737**	**789.39**	**0.0163**	**21**	**355**	**361**	**EPDATTR**	
**797.39**	**797.3966**	**0.0066**	**8**	**55**	**60**	**ENHELR**	
**859.4631**	**859.4672**	**0.0041**	**5**	**229**	**237**	**GLAGISGER**	
**863.4257**	**863.4281**	**0.0024**	**3**	**284**	**291**	**NVDGFSPK**	

936.4785	936.4796	0.0011	1	191	197	IQEDYLR	
1038.5037	1038.5056	0.0019	2	91	99	EMFQSAGIR	
1054.4987	1054.4928	-0.0059	-6	91	99	EMFQSAGIR	Oxidation (M)[2]
1147.5564	1147.5369	-0.0195	-17	368	376	YQGEHCLLK	Carbamidomethyl (C)[6]
1490.691	1490.677	-0.014	-9	135	146	HAEVEFTTDWQK	
1501.7645	1501.7484	-0.0161	-11	115	126	LHEENFEITTLR	

Spot #30. MALDI MS data - 42% & 32% coverage

**Gel Idx/Pos**	32/B11	**Instr./Gel Origin**	gloin/Spotset050705	**Process Status**	Analysis Succeeded
**Plate [#] Name**	[1] Sandrine	**Instrument Sample Name**		**Spectra**	1

Rank	Protein Name	Accession No.	Protein Score	Protein Score C. I. %	Total Ion C. I. %	Protein MW	Protein PI	Pep. Count	Intensity Matched
1	(P52597) Heterogeneous nuclear ribonucleoprotein F (hnRNP F) (Nucleolin-like protein mcs94-1)	HNRPF_HUMAN	157	100		45853.9	5.38	16	11.861

Peptide Information

Calc. Mass	Obsrv. Mass	± da	± ppm	Start Seq.	End Seq.	Sequence	Modification
732.3093	732.3191	0.0098	13	75	80	ESMGHR	Oxidation (M)[3]
756.3569	756.3563	-0.0006	-1	349	354	ANMQHR	
767.3868	767.3907	0.0039	5	200	205	FMSVQR	
772.3519	772.3477	-0.0042	-5	349	354	ANMQHR	Oxidation (M)[3]
783.3817	783.3774	-0.0043	-5	200	205	FMSVQR	Oxidation (M)[2]
798.4395	798.4313	-0.0082	-10	179	184	YIEVFK	
834.3951	834.4037	0.0086	10	185	191	SSQEEVR	
879.4392	879.4285	-0.0107	-12	116	123	GLPFGCTK	Carbamidomethyl (C)[6]
906.4567	906.449	-0.0077	-8	192	199	SYSDPPLK	
1021.5023	1021.4971	-0.0052	-5	90	97	TEMDWVLK	
1037.4972	1037.4874	-0.0098	-9	90	97	TEMDWVLK	Oxidation (M)[3]
1092.5796	1092.5718	-0.0078	-7	316	325	VHIEIGPDGR	
1361.6769	1361.6605	-0.0164	-12	1	13	MLGPEGGEGFVVK	N-Acetyl (Protein)[0]
1377.6719	1377.6575	-0.0144	-10	1	13	MLGPEGGEGFVVK	N-Acetyl (Protein)[0], Oxidation (M)[1]
1616.6832	1616.677	-0.0062	-4	262	274	DLSYCLSGMYDHR	Carbamidomethyl (C)[5]
1630.7205	1630.7153	-0.0052	-3	98	113	HSGPNSADSANDGFVR	
1709.7864	1709.7701	-0.0163	-10	52	67	QSGEAFVELGSEDDVK	
1867.9436	1867.9335	-0.0101	-5	150	166	ITGEAFVQFASQELAEK	
1996.9763	1996.9783	0.002	1	299	315	ATENDIYNFFSPLNPVR	
2208.0125	2208.0056	-0.0069	-3	326	346	VTGEADVEFATHEEAVAAMSK	Oxidation (M)[19]

2	(O00303) Eukaryotic translation initiation factor 3 subunit 5 (eIF-3 epsilon) (eIF3 p47 subunit) (e	IF35_HUMAN	88	99.998	37654.2	5.24	10	9.791

Peptide Information

Calc. Mass	Obsrv. Mass	± da	± ppm	Start Seq.	End Seq.	Sequence	Modification
881.3934	881.3935	0.0001	0	255	261	TCFSPNR	Carbamidomethyl (C)[2]
918.4639	918.4584	-0.0055	-6	298	306	VSADNTVGR	
934.4451	934.4431	-0.002	-2	155	161	NMYELHK	
950.44	950.4297	-0.0103	-11	155	161	NMYELHK	Oxidation (M)[2]
1080.4633	1080.4606	-0.0027	-2	239	246	YAYYDTER	
1250.6561	1250.6473	-0.0088	-7	215	226	AYVSTLMGVPGR	
1266.6511	1266.6409	-0.0102	-8	215	226	AYVSTLMGVPGR	Oxidation (M)[7]
1291.7079	1291.6838	-0.0241	-19	307	317	FLMSLVNQVPK	Oxidation (M)[3]
1340.6953	1340.6639	-0.0314	-23	227	238	TMGVMFTPLTVK	Oxidation (M)[2]
1356.6902	1356.6769	-0.0133	-10	227	238	TMGVMFTPLTVK	Oxidation (M)[2,5]
1657.8867	1657.8787	-0.008	-5	262	278	VIGLSSDLQQVGGASAR	
1711.9377	1711.9263	-0.0114	-7	94	108	LHPVILASIVDSYER	
1962.0039	1961.9896	-0.0143	-7	193	210	EAPNPIHLTVDTSLQNGR	

Spot #31. MALDI MS data - 37% coverage

**Gel Idx/Pos**	104/E7	**Instr./Gel Origin**	gloin/Spotset041117	**Process Status**	Analysis Succeeded
**Plate [#] Name**	[1] Sandrine	**Instrument Sample Name**		**Spectra**	1

Rank	Protein Name	Accession No.	Protein Score	Protein Score C. I. %	Total Ion C. I. %	Protein MW	Protein PI	Pep. Count	Intensity Matched
1	(P04181) Ornithine aminotransferase, mitochondrial precursor (EC 2.6.1.13) (Ornithine--oxo-acid ami	OAT_HUMAN	124	100		48846.3	6.57	14	9.382

Peptide Information

Calc. Mass	Obsrv. Mass	± da	± ppm	Start Seq.	End Seq.	Sequence	Modification
820.3624	820.3621	-0.0003	0	387	392	DWDAWK	
821.4304	821.4288	-0.0016	-2	130	135	LFNYHK	
884.5603	884.5515	-0.0088	-10	414	421	FAPPLVIK	
908.4948	908.4919	-0.0029	-3	406	413	PTHGDIIR	
945.5251	945.5191	-0.006	-6	427	434	ESIEIINK	
1056.6047	1056.6034	-0.0013	-1	363	372	LPSDVVTAVR	
1207.6106	1207.6108	0.0002	0	67	76	GIYLWDVEGR	
1237.6476	1237.6409	-0.0067	-5	170	180	IVFAAGNFWGR	
1561.7493	1561.7484	-0.0009	-1	33	46	TVQGPPTSDDIFER	
1692.7931	1692.8124	0.0193	11	136	151	VLPMNTGVEAGETACK	Carbamidomethyl (C)[15], Oxidation (M)[4]
1736.8867	1736.885	-0.0017	-1	50	64	YGAHNYHPLPVALER	
1810.981	1810.9767	-0.0043	-2	256	271	HQVLFIADEIQTGLAR	
1952.9276	1952.9156	-0.012	-6	114	129	AFYNNVLGEYEEYITK	
2141.0972	2141.0994	0.0022	1	332	351	VAIAALEVLEEENLAENADK	

Spot #32. MALDI MS data - 35% coverage

**Gel Idx/Pos**	117/E10	**Instr./Gel Origin**	gloin/Spotset040318	**Process Status**	Analysis Succeeded
**Plate [#] Name**	[1] Sandrine	**Instrument Sample Name**		**Spectra**	1

Rank	Protein Name	Accession No.	Protein Score	Protein Score C. I. %	Total Ion C. I. %	Protein MW	Protein PI	Pep. Count	Intensity Matched
1	(Q9P2R7) Succinyl-CoA ligase [ADP-forming] beta-chain, mitochondrial precursor (EC 6.2.1.5) (Succin	SUCB1_HUMAN	129	100		50641.4	7.05	16	8.123

Peptide Information

Calc. Mass	Obsrv. Mass	± da	± ppm	Start Seq.	End Seq.	Sequence	Modification
771.4471	771.4473	0.0002	0	99	106	AQVLAGGR	
883.5287	883.5237	-0.005	-6	243	249	LYSLFLK	
887.5196	887.5111	-0.0085	-10	418	426	ALIADSGLK	
936.4818	936.4811	-0.0007	-1	130	138	AVSSQMIGK	Oxidation (M)[6]
973.5928	973.5781	-0.0147	-15	443	451	LSEIVTLAK	
1019.5407	1019.5388	-0.0019	-2	121	129	IVFSPEEAK	
1028.5735	1028.5684	-0.0051	-5	217	225	EQALQLAQK	
1064.5259	1064.5244	-0.0015	-1	79	88	SPDEAYAIAK	
1142.594	1142.5848	-0.0092	-8	206	215	EPIDIEEGIK	
1186.5487	1186.5538	0.0051	4	275	284	INFDSNSAYR	
1290.6293	1290.6259	-0.0034	-3	151	160	ICNQVLVCER	Carbamidomethyl (C)[2,8]
1322.6085	1322.6177	0.0092	7	166	175	EYYFAITMER	
1338.6034	1338.5975	-0.0059	-4	166	175	EYYFAITMER	Oxidation (M)[8]
1361.6366	1361.6342	-0.0024	-2	427	438	ILACDDLDEAAR	Carbamidomethyl (C)[4]
1419.7334	1419.7183	-0.0151	-11	384	396	CDVIAQGIVMAVK	Carbamidomethyl (C)[1], Oxidation (M)[10]
1694.7656	1694.7744	0.0088	5	288	300	IFDLQDWTQEDER	
1865.8772	1865.8645	-0.0127	-7	226	242	MGFPPNIVESAAENMVK	Oxidation (M)[1,15]

Spot #33. MALDI MS data - 43% coverage

**Gel Idx/Pos**	128/F7	**Instr./Gel Origin**	gloin/Spotset040318	**Process Status**	Analysis Succeeded
**Plate [#] Name**	[1] Sandrine	**Instrument Sample Name**		**Spectra**	1

Rank	Protein Name	Accession No.	Protein Score	Protein Score C. I. %	Total Ion C. I. %	Protein MW	Protein PI	Pep. Count	Intensity Matched
1	(P51570) Galactokinase (EC 2.7.1.6) (Galactose kinase)	GALK1_HUMAN	141	100		42701.7	6.04	15	9.522

Peptide Information

Calc. Mass	Obsrv. Mass	± da	± ppm	Start Seq.	End Seq.	Sequence	Modification
780.4039	780.3965	-0.0074	-9	106	111	WANYVK	
809.4628	809.4551	-0.0077	-10	280	286	HVVGEIR	
872.4948	872.5015	0.0067	8	288	296	TAQAAAALR	
887.4403	887.4341	-0.0062	-7	306	312	LMVESHR	Oxidation (M)[2]
891.3988	891.4193	0.0205	23	242	248	QCEEVAR	Carbamidomethyl (C)[2]
1054.5463	1054.5492	0.0029	3	196	204	GHALLIDCR	Carbamidomethyl (C)[8]
1170.663	1170.6661	0.0031	3	88	97	LQFPLPTAQR	
1199.7106	1199.7078	-0.0028	-2	218	228	LAVLITNSNVR	
1245.6222	1245.6232	0.001	1	229	239	HSLASSEYPVR	
1286.6587	1286.6597	0.001	1	257	267	EVQLEELEAAR	
1324.7219	1324.7235	0.0016	1	6	17	QPQVAELLAEAR	
1385.7522	1385.7277	-0.0245	-18	205	217	SLETSLVPLSDPK	
1658.802	1658.8063	0.0043	3	22	37	EEFGAEPELAVSAPGR	
1887.9294	1887.9331	0.0037	2	70	87	DGLVSLLTTSEGADEPQR	
2365.1206	2365.127	0.0064	3	367	388	HIQEHYGGTATFYLSQAADGAK	

Spot #34. MALDI MS data - 36% coverage

**Gel Idx/Pos**	105/E9	**Instr./Gel Origin**	gloin/Spotset041117	**Process Status**	Analysis Succeeded
**Plate [#] Name**	[1] Sandrine	**Instrument Sample Name**		**Spectra**	1

Rank	Protein Name	Accession No.	Protein Score	Protein Score C. I. %	Total Ion C. I. %	Protein MW	Protein PI	Pep. Count	Intensity Matched
1	(Q15019) Septin-2 (Protein NEDD5)	SEPT2_HUMAN	95	100		41689.3	6.15	12	8.876

Peptide Information

Calc. Mass	Obsrv. Mass	± da	± ppm	Start Seq.	End Seq.	Sequence	Modification
767.4158	767.4114	-0.0044	-6	140	145	HIIDNR	
784.4562	784.4398	-0.0164	-21	67	74	VIPGAAEK	
858.493	858.4929	-0.0001	0	319	325	DQILLEK	
910.412	910.4052	-0.0068	-7	332	338	MQEMIAR	Oxidation (M)[1,4]
952.6189	952.6165	-0.0024	-3	175	183	VNIVPVIAK	
1203.5753	1203.5718	-0.0035	-3	129	138	YLHDESGLNR	
1352.7056	1352.6919	-0.0137	-10	199	209	ILDEIEEHNIK	
1513.7533	1513.7429	-0.0104	-7	117	128	TIISYIDEQFER	
1603.8174	1603.8097	-0.0077	-5	78	91	TVQIEASTVEIEER	
1750.8429	1750.8381	-0.0048	-3	97	112	LTVVDTPGYGDAINCR	Carbamidomethyl (C)[15]
1759.9589	1759.9506	-0.0083	-5	233	249	ASIPFSVVGSNQLIEAK	
1881.9956	1881.9817	-0.0139	-7	51	66	STLINSLFLTDLYPER	

Spot #35. MALDI MS data - 44% coverage

**Gel Idx/Pos**	150/G3	**Instr./Gel Origin**	gloin/Spotset040318	**Process Status**	Analysis Succeeded
**Plate [#] Name**	[1] Sandrine	**Instrument Sample Name**		**Spectra**	1

Rank	Protein Name	Accession No.	Protein Score	Protein Score C. I. %	Total Ion C. I. %	Protein MW	Protein PI	Pep. Count	Intensity Matched
1	(O00154) Cytosolic acyl coenzyme A thioester hydrolase (EC 3.1.2.2) (Long chain acyl-CoA thioester	BACH_HUMAN	130	100		42453.6	8.85	14	9.955

Peptide Information

Calc. Mass	Obsrv. Mass	± da	± ppm	Start Seq.	End Seq.	Sequence	Modification
760.4133	760.4102	-0.0031	-4	100	106	CVAALAR	Carbamidomethyl (C)[1]
828.392	828.3918	-0.0002	0	296	302	MTFTSNK	
863.4006	863.4109	0.0103	12	373	380	QGHAEPQP	
962.5087	962.5129	0.0042	4	287	295	GCVITISGR	Carbamidomethyl (C)[2]
1004.4279	1004.4296	0.0017	2	184	191	QEQEEEGR	
1101.449	1101.4542	0.0052	5	91	99	HCNSQNGER	Carbamidomethyl (C)[2]
1244.6667	1244.6715	0.0048	4	253	264	LMDEVAGIVAAR	
1257.7201	1257.724	0.0039	3	173	183	VLEVPPVVYSR	
1260.6616	1260.6649	0.0033	3	253	264	LMDEVAGIVAAR	Oxidation (M)[2]
1290.6722	1290.6588	-0.0134	-10	79	90	MIEEAGAIISTR	
1306.6671	1306.667	-0.0001	0	79	90	MIEEAGAIISTR	Oxidation (M)[1]
1680.869	1680.8652	-0.0038	-2	341	355	SLPVPQLVPETEDEK	
1733.8492	1733.8514	0.0022	1	325	340	AASAFFTYVSLSQEGR	
1744.8865	1744.8771	-0.0094	-5	268	283	TNIVTASVDAINFHDK	
2061.0056	2061.002	-0.0036	-2	303	321	SMEIEVLVDADPVVDSSQK	
2071.0488	2071.0593	0.0105	5	134	152	HSVEVQVNVMSENILTGAK	Oxidation (M)[10]
2077.0005	2077.0066	0.0061	3	303	321	SMEIEVLVDADPVVDSSQK	Oxidation (M)[2]

Spot #36. MALDI MS data - 26% coverage

**Gel Idx/Pos**	38/B23	**Instr./Gel Origin**	gloin/Spotset050705	**Process Status**	Analysis Succeeded
**Plate [#] Name**	[1] Sandrine	**Instrument Sample Name**		**Spectra**	1

Rank	Protein Name	Accession No.	Protein Score	Protein Score C. I. %	Total Ion C. I. %	Protein MW	Protein PI	Pep. Count	Intensity Matched
1	(Q08752) 40 kDa peptidyl-prolyl cis-trans isomerase (EC 5.2.1.8) (PPIase) (Rotamase) (Cyclophilin-4	PPID_HUMAN	86	99.997		41006	6.76	12	3.711

Peptide Information

Calc. Mass	Obsrv. Mass	± da	± ppm	Start Seq.	End Seq.	Sequence	Modification
737.3576	737.3627	0.0051	7	43	48	TAENFR	
750.4144	750.4165	0.0021	3	245	250	YAEVLR	
770.4155	770.4382	0.0227	29	56	63	GIGHTTGK	
773.3511	773.358	0.0069	9	69	74	GCPFHR	Carbamidomethyl (C)[2]
868.4271	868.4335	0.0064	7	9	16	PSNPSNPR	
887.4733	887.4813	0.008	9	313	320	AQGWQGLK	
893.4475	893.4473	-0.0002	0	1	8	SHPSPQAK	N-Acetyl (Protein)[0]
940.4886	940.481	-0.0076	-8	227	234	NIGNTFFK	
1106.5299	1106.5237	-0.0062	-6	235	243	SQNWEMAIK	
1165.5735	1165.5637	-0.0098	-8	321	330	EYDQALADLK	
1228.5269	1228.512	-0.0149	-12	102	110	FEDENFHYK	
1253.6161	1253.6044	-0.0117	-9	17	27	VFFDVDIGGER	

Spot #37. MALDI MS data - 26% coverage

Gel Idx/Pos	127/F5	Instr./Gel Origin	gloin/Spotset040318	Process Status	Analysis Succeeded
Plate [#] Name	[1] Sandrine	Instrument Sample Name		Spectra	1

Rank	Protein Name	Accession No.	Protein Score	Protein Score C. I. %	Total Ion C. I. %	Protein MW	Protein PI	Pep. Count	Intensity Matched
1	(O43681) Arsenical pump-driving ATPase (EC 3.6.3.16) (Arsenite-translocating ATPase) (Arsenical res	ARSA1_HUMAN	61	99.012		39223.6	4.81	8	6.325

Peptide Information

Calc. Mass	Obsrv. Mass	± da	± ppm	Start Seq.	End Seq.	Sequence	Modification
806.4559	806.4461	-0.0098	-12	39	45	WIFVGGK	
814.5032	814.4979	-0.0053	-7	261	267	LIQELAK	
1069.6252	1069.6248	-0.0004	0	222	230	LEETLPVIR	
1073.6466	1073.6471	0.0005	0	318	326	LPLLPHEVR	
1201.694	1201.692	-0.002	-2	176	185	LLNFPTIVER	
1454.6978	1454.6947	-0.0031	-2	51	63	TTCSCSLAVQLSK	Carbamidomethyl (C)[3,5]
2073.9473	2073.9373	-0.01	-5	302	317	YLDQMEDLYEDFHIVK	Oxidation (M)[5]
2300.1404	2300.1455	0.0051	2	66	86	ESVLIISTDPAHNISDAFDQK	

Spot #38. MALDI MS data - 48% coverage

**Gel Idx/Pos**	29/B5	**Instr./Gel Origin**	gloin/Spotset050705	**Process Status**	Analysis Succeeded
**Plate [#] Name**	[1] Sandrine	**Instrument Sample Name**		**Spectra**	1

Rank	Protein Name	Accession No.	Protein Score	Protein Score C. I. %	Total Ion C. I. %	Protein MW	Protein PI	Pep. Count	Intensity Matched
1	(Q9Y3F4) Serine-threonine kinase receptor-associated protein (UNR-interacting protein) (WD-40 repea	STRAP_HUMAN	161	100		38756.1	4.98	14	7.375

Peptide Information

Calc. Mass	Obsrv. Mass	± da	± ppm	Start Seq.	End Seq.	Sequence	Modification
767.4086	767.3947	-0.0139	-18	299	304	TYGLWK	
930.5406	930.5255	-0.0151	-16	291	298	LWQTVVGK	
1008.4455	1008.4336	-0.0119	-12	149	156	ALWCSEDK	Carbamidomethyl (C)[4]
1016.5522	1016.5383	-0.0139	-14	60	69	GAVWGATLNK	
1028.5371	1028.5237	-0.0134	-13	138	147	EISGHTSGIK	
1179.584	1179.5854	0.0014	1	263	272	GHFGPIHCVR	Carbamidomethyl (C)[8]
1211.5942	1211.5858	-0.0084	-7	236	246	EFLVAGGEDFK	
1346.641	1346.634	-0.007	-5	168	178	LWDHATMTEVK	Oxidation (M)[7]
1484.8108	1484.798	-0.0128	-9	205	218	SIAFHSAVSLDPIK	
1596.67	1596.6608	-0.0092	-6	250	262	YDYNSGEELESYK	
1787.8633	1787.8727	0.0094	5	85	100	VWDAVSGDELMTLAHK	Oxidation (M)[11]
1798.897	1798.8909	-0.0061	-3	219	235	SFEAPATINSASLHPEK	
1900.856	1900.8556	-0.0004	0	273	290	FSPDGELYASGSEDGTLR	
2001.9399	2001.9336	-0.0063	-3	105	122	TVDFTQDSNYLLTGGQDK	

Spot #39. MALDI MS data - 35% coverage

**Gel Idx/Pos**	22/A16	**Instr./Gel Origin**	gloin/Spotset050705	**Process Status**	Analysis Succeeded
**Plate [#] Name**	[1] Sandrine	**Instrument Sample Name**		**Spectra**	1

Rank	Protein Name	Accession No.	Protein Score	Protein Score C. I. %	Total Ion C. I. %	Protein MW	Protein PI	Pep. Count	Intensity Matched
1	(Q9UBE0) Ubiquitin-like 1-activating enzyme E1A (SUMO-1-activating enzyme subunit 1)	ULE1A_HUMAN	98	100		38881.7	5.17	12	6.634

Peptide Information

Calc. Mass	Obsrv. Mass	± da	± ppm	Start Seq.	End Seq.	Sequence	Modification
775.3944	775.3969	0.0025	3	92	98	AEASLER	
827.5349	827.5204	-0.0145	-18	54	61	NLILAGVK	
848.4698	848.4599	-0.0099	-12	211	217	VVFCPVK	Carbamidomethyl (C)[4]
899.4403	899.4396	-0.0007	-1	142	148	VDQICHK	Carbamidomethyl (C)[5]
944.52	944.5304	0.0104	11	25	32	LWGLEAQK	
948.452	948.4637	0.0117	12	110	117	VDTEDIEK	
1126.5297	1126.5455	0.0158	14	199	208	LDSSETTMVK	Oxidation (M)[8]
1143.5826	1143.5721	-0.0105	-9	336	346	GNGIVECLGPK	Carbamidomethyl (C)[7]
1228.6355	1228.6301	-0.0054	-4	99	109	AQNLNPMVDVK	
1244.6304	1244.619	-0.0114	-9	99	109	AQNLNPMVDVK	Oxidation (M)[7]
1292.6005	1292.5938	-0.0067	-5	218	228	EALEVDWSSEK	
1810.7726	1810.7743	0.0017	1	5	21	EEAGGGISEEEAAQYDR	
2109.9822	2110.0073	0.0251	12	256	273	DPSSDTYEEDSELLLQIR	

Spot #40. MALDI MS data - 37% coverage

**Gel Idx/Pos**	107/E13	**Instr./Gel Origin**	gloin/Spotset041117	**Process Status**	Analysis Succeeded
**Plate [#] Name**	[1] Sandrine	**Instrument Sample Name**		**Spectra**	1

Rank	Protein Name	Accession No.	Protein Score	Protein Score C. I. %	Total Ion C. I. %	Protein MW	Protein PI	Pep. Count	Intensity Matched
1	(P04899) Guanine nucleotide-binding protein G(i), alpha-2 subunit (Adenylate cyclase-inhibiting G a	GNAI2_HUMAN	97	100		40864.2	5.34	11	6.924

Peptide Information

Calc. Mass	Obsrv. Mass	± da	± ppm	Start Seq.	End Seq.	Sequence	Modification
780.3774	780.3668	-0.0106	-14	272	277	DLFEEK	
909.4247	909.4287	0.004	4	198	205	MFDVGGQR	
925.4196	925.4138	-0.0058	-6	198	205	MFDVGGQR	Oxidation (M)[1]
1110.5248	1110.5153	-0.0095	-9	249	257	LFDSICNNK	Carbamidomethyl (C)[6]
1274.59	1274.5793	-0.0107	-8	296	306	YDEAASYIQSK	
1380.7158	1380.7061	-0.0097	-7	181	192	TTGIVETHFTFK	
1516.7114	1516.7004	-0.011	-7	130	142	LWADHGVQACFGR	Carbamidomethyl (C)[10]
1534.7318	1534.7308	-0.001	-1	86	99	AMGNLQIDFADPSR	
1550.7268	1550.7128	-0.014	-9	86	99	AMGNLQIDFADPSR	Oxidation (M)[2]
1586.7156	1586.7164	0.0008	1	318	330	EIYTHFTCATDTK	Carbamidomethyl (C)[8]
1636.6908	1636.6837	-0.0071	-4	54	66	IIHEDGYSEEECR	Carbamidomethyl (C)[12]
1746.9021	1746.8956	-0.0065	-4	162	176	IAQSDYIPTQQDVLR	
2076.9509	2076.948	-0.0029	-1	145	161	EYQLNDSAAYYLNDLER	

Spot #41. MALDI MS data - 30 & 30% coverage

**Gel Idx/Pos**	125/F1	**Instr./Gel Origin**	gloin/Spotset040318	**Process Status**	Analysis Succeeded
**Plate [#] Name**	[1] Sandrine	**Instrument Sample Name**		**Spectra**	1

Rank	Protein Name	Accession No.	Protein Score	Protein Score C. I. %	Total Ion C. I. %	Protein MW	Protein PI	Pep. Count	Intensity Matched
1	(Q15257) Protein phosphatase 2A, regulatory subunit B' (PP2A, subunit B', PR53 isoform) (Phosphotyr	PTPA_HUMAN	96	100		41111.6	5.63	12	8.079

Peptide Information

Calc. Mass	Obsrv. Mass	± da	± ppm	Start Seq.	End Seq.	Sequence	Modification
749.3498	749.3669	0.0171	23	332	337	AECLEK	Carbamidomethyl (C)[3]
774.378	774.3802	0.0022	3	272	277	HFVDEK	
799.4784	799.4798	0.0014	2	322	328	VNQGLIR	
810.4178	810.4202	0.0024	3	223	228	YLEVMR	
811.4056	811.415	0.0094	12	278	284	AVNENHK	
826.4127	826.4101	-0.0026	-3	223	228	YLEVMR	Oxidation (M)[5]
828.425	828.4264	0.0014	2	64	69	LTFEYR	
849.406	849.4083	0.0023	3	176	183	ESVGNSTR	
1015.5723	1015.5657	-0.0066	-6	338	345	FPVIQHFK	
1126.5562	1126.5343	-0.0219	-19	29	38	EIHTVPDMGK	
1142.551	1142.5378	-0.0132	-12	29	38	EIHTVPDMGK	Oxidation (M)[8]
1147.6357	1147.6334	-0.0023	-2	209	218	VDDQIAIVFK	
1539.7438	1539.7504	0.0066	4	122	134	WIDETPPVDQPSR	
2263.124	2263.1184	-0.0056	-2	7	27	QPPPDSSEEAPPATQNFIIPK	

2	(Q15417) Calponin-3 (Calponin, acidic isoform)	CNN3_HUMAN	76	99.964	36561.9	5.69	10	5.699

Peptide Information

Calc. Mass	Obsrv. Mass	± da	± ppm	Start Seq.	End Seq.	Sequence	Modification
749.3828	749.3669	-0.0159	-21	266	271	QVYDPK	
772.3987	772.3994	0.0007	1	187	192	HLYDPK	
781.3726	781.3696	-0.003	-4	227	232	DIYDQK	
955.4665	955.4636	-0.0029	-3	257	265	GMSVYGLGR	Oxidation (M)[2]
1107.568	1107.5618	-0.0062	-6	7	17	GPSYGLSAEVK	
1187.6418	1187.6331	-0.0087	-7	133	143	GFHTTIDIGVK	
1216.6102	1216.6111	0.0009	1	213	225	GASQAGMLAPGTR	
1232.6052	1232.6088	0.0036	3	213	225	GASQAGMLAPGTR	Oxidation (M)[7]
1275.5601	1275.5613	0.0012	1	24	33	YDHQAEEDLR	
1373.5936	1373.6052	0.0116	8	173	185	CASQAGMTAYGTR	Carbamidomethyl (C)[1]
1389.5886	1389.5906	0.002	1	173	185	CASQAGMTAYGTR	Carbamidomethyl (C)[1], Oxidation (M)[7]
1419.7261	1419.7273	0.0012	1	159	172	AGQSVIGLQMGTNK	Oxidation (M)[10]

Spot #42. MALDI MS data - 44% coverage

**Gel Idx/Pos**	160/G23	**Instr./Gel Origin**	gloin/Spotset040318	**Process Status**	Analysis Succeeded
**Plate [#] Name**	[1] Sandrine	**Instrument Sample Name**		**Spectra**	1

Rank	Protein Name	Accession No.	Protein Score	Protein Score C. I. %	Total Ion C. I. %	Protein MW	Protein PI	Pep. Count	Intensity Matched
1	(P53004) Biliverdin reductase A precursor (EC 1.3.1.24) (Biliverdin-IX alpha-reductase) (BVR A)	BIEA_HUMAN	119	100		33692.4	6.06	13	12.665

Peptide Information

Calc. Mass	Obsrv. Mass	± da	± ppm	Start Seq.	End Seq.	Sequence	Modification
745.2756	745.2767	0.0011	1	291	295	YCCSR	Carbamidomethyl (C)[2,3]
777.4253	777.4175	-0.0078	-10	86	92	QFLNAGK	
921.504	921.5022	-0.0018	-2	262	269	LLGQFSEK	
941.4879	941.4875	-0.0004	0	228	234	YLSFHFK	
988.5937	988.5922	-0.0015	-2	9	18	FGVVVVGVGR	
991.5206	991.5179	-0.0027	-3	254	261	DQNIFVQK	
1088.5623	1088.5574	-0.0049	-5	211	219	SPLSWIEEK	
1110.517	1110.5312	0.0142	13	201	209	MTVCLETEK	Carbamidomethyl (C)[4]
1126.5118	1126.5419	0.0301	27	201	209	MTVCLETEK	Carbamidomethyl (C)[4], Oxidation (M)[1]
1185.6051	1185.6123	0.0072	6	162	172	FGFPAFSGISR	
1413.7332	1413.7411	0.0079	6	235	248	SGSLENVPNVGVNK	
1518.7798	1518.787	0.0072	5	148	161	GSLLFTAGPLEEER	
1523.8251	1523.8243	-0.0008	-1	278	290	ILHCLGLAEEIQK	Carbamidomethyl (C)[4]
1855.9813	1855.9673	-0.014	-8	29	45	NPHPSSAFLNLIGFVSR	

Spot #43. ESI MS/MS data

THTM_HUMAN Mass: 33312 Score: 437 Queries matched: 12
(P25325) 3-mercaptopyruvate sulfurtransferase (EC 2.8.1.2) (MST)

Check to include this hit in error tolerant search or archive report

Query	Observed	Mr(expt)	Mr(calc)	Delta	Miss	Score	Expect	Rank	Peptide
5	306,16	610,3	610,33	-0,03	0	16	2,6	3	HWLR
18	401,19	800,37	800,42	-0,05	0	24	0,93	1	HLFQEK
22	425,71	849,4	849,43	-0,03	0	34	0,073	1	FQVVDSR
26	433,19	864,37	864,41	-0,04	1	29	0,23	3	REFEER
28	437,19	872,37	872,42	-0,05	0	43	0,013	1	ASPQLCR + N-Acetyl (Protein)
33	472,23	942,45	942,51	-0,07	0	53	0,0011	1	QNLPLSSGK
36	501,72	1001,43	1001,49	-0,06	0	37	0,041	1	SQPAPAEFR
37	501,76	1001,51	1001,55	-0,04	0	30	0,23	1	AQLDPAFIK
38	503,74	1005,46	1005,54	-0,08	1	29	0,26	1	RFQVVDSR
40	544,73	1087,45	1087,54	-0,1	1	43	0,0098	1	FRGTEPEPR
41	614,78	1227,55	1227,62	-0,07	1	52	0,0014	1	ARPEDVISEGR
43	499,54	1495,61	1495,72	-0,11	1	51	0,0015	1	TYEDIKENLESR

Spot #44. MALDI MS data - 32% coverage

**Gel Idx/Pos**	122/E20	**Instr./Gel Origin**	gloin/Spotset040318	**Process Status**	Analysis Succeeded
**Plate [#] Name**	[1] Sandrine	**Instrument Sample Name**		**Spectra**	1

Rank	Protein Name	Accession No.	Protein Score	Protein Score C. I. %	Total Ion C. I. %	Protein MW	Protein PI	Pep. Count	Intensity Matched
1	(P40925) Malate dehydrogenase, cytoplasmic (EC 1.1.1.37)	MDHC_HUMAN	85	99.996		36500.1	6.89	11	11.186

Peptide Information

Calc. Mass	Obsrv. Mass	± da	± ppm	Start Seq.	End Seq.	Sequence	Modification
763.3621	763.3587	-0.0034	-4	214	219	DDSWLK	
789.41	789.4043	-0.0057	-7	110	117	SQGAALDK	
870.425	870.4237	-0.0013	-1	248	254	AICDHVR	Carbamidomethyl (C)[3]
916.5098	916.5027	-0.0071	-8	170	178	LGVTANDVK	
1007.5407	1007.5385	-0.0022	-2	205	213	EVGVYEALK	
1026.4673	1026.4708	0.0035	3	149	156	ENFSCLTR	Carbamidomethyl (C)[5]
1087.4943	1087.4932	-0.0011	-1	324	333	ESAFEFLSSA	
1164.6007	1164.6045	0.0038	3	220	229	GEFVTTVQQR	
1393.7111	1393.7153	0.0042	3	298	309	FVEGLPINDFSR	
1401.7406	1401.743	0.0024	2	79	91	DLDVAILVGSMPR	Oxidation (M)[11]
1757.9214	1757.9233	0.0019	1	125	141	VIVVGNPANTNCLTASK	Carbamidomethyl (C)[12]

Spot #45. MALDI MS data - 45% coverage

**Gel Idx/Pos**	110/E19	**Instr./Gel Origin**	gloin/Spotset041117	**Process Status**	Analysis Succeeded
**Plate [#] Name**	[1] Sandrine	**Instrument Sample Name**		**Spectra**	1

Rank	Protein Name	Accession No.	Protein Score	Protein Score C. I. %	Total Ion C. I. %	Protein MW	Protein PI	Pep. Count	Intensity Matched
1	(P54920) Alpha-soluble NSF attachment protein (SNAP-alpha) (N-ethylmaleimide-sensitive factor attac	SNAA_HUMAN	111	100		33681.5	5.23	11	13.005

Peptide Information

Calc. Mass	Obsrv. Mass	± da	± ppm	Start Seq.	End Seq.	Sequence	Modification
869.3999	869.3983	-0.0016	-2	265	271	EYDSISR	
1168.5667	1168.5653	-0.0014	-1	107	116	AIEIYTDMGR	
1175.5538	1175.551	-0.0028	-2	286	295	TIQGDEEDLR	
1184.5616	1184.5585	-0.0031	-3	107	116	AIEIYTDMGR	Oxidation (M)[8]
1253.583	1253.5745	-0.0085	-7	38	47	IEEACEIYAR	Carbamidomethyl (C)[5]
1403.6835	1403.678	-0.0055	-4	7	19	EAEAMALLAEAER	
1417.6562	1417.6547	-0.0015	-1	95	106	ADPQEAINCLMR	Carbamidomethyl (C)[9]
1419.6783	1419.6713	-0.007	-5	7	19	EAEAMALLAEAER	Oxidation (M)[5]
1433.6511	1433.6584	0.0073	5	95	106	ADPQEAINCLMR	Carbamidomethyl (C)[9], Oxidation (M)[11]
1453.7686	1453.766	-0.0026	-2	168	180	VAGYAALLEQYQK	
1460.6692	1460.6689	-0.0003	0	228	239	YEELFPAFSDSR	
1558.7173	1558.7194	0.0021	1	141	153	AIAHYEQSADYYK	
1623.722	1623.7206	-0.0014	-1	79	93	HDAATCFVDAGNAFK	Carbamidomethyl (C)[6]
2090.9875	2090.9856	-0.0019	-1	247	264	LLEAHEEQNVDSYTESVK	

Spot #46. MALDI MS data - 70% coverage

**Gel Idx/Pos**	165/G10	**Instr./Gel Origin**	gloin/Spotset040318	**Process Status**	Analysis Succeeded
**Plate [#] Name**	[1] Sandrine	**Instrument Sample Name**		**Spectra**	1

Rank	Protein Name	Accession No.	Protein Score	Protein Score C. I. %	Total Ion C. I. %	Protein MW	Protein PI	Pep. Count	Intensity Matched
1	(P08758) Annexin A5 (Annexin V) (Lipocortin V) (Endonexin II) (Calphobindin I) (CBP-I) (Placental a	ANXA5_HUMAN	215	100		35840.4	4.94	19	21.857

Peptide Information

Calc. Mass	Obsrv. Mass	± da	± ppm	Start Seq.	End Seq.	Sequence	Modification
744.3886	744.3875	-0.0011	-1	117	122	TPEELR	
775.3944	775.3902	-0.0042	-5	18	24	ADAETLR	
831.4458	831.4461	0.0003	0	63	69	DLLDDLK	
864.3734	864.3696	-0.0038	-4	186	192	WGTDEEK	
893.4727	893.4691	-0.0036	-4	50	57	QEISAAFK	
954.5406	954.5408	0.0002	0	193	200	FITIFGTR	
1001.5989	1001.5972	-0.0017	-2	108	116	VLTEIIASR	
1014.5142	1014.5106	-0.0036	-4	89	96	LYDAYELK	
1106.584	1106.583	-0.001	-1	276	284	SEIDLFNIR	
1155.5753	1155.5787	0.0034	3	260	270	GAGTDDHTLIR	
1172.682	1172.6799	-0.0021	-2	151	160	MLVVLLQANR	Oxidation (M)[1]
1290.6399	1290.6395	-0.0004	0	290	300	NFATSLYSMIK	Oxidation (M)[9]
1340.6117	1340.6125	0.0008	1	6	17	GTVTDFPGFDER	
1613.9108	1613.9088	-0.002	-1	227	241	ETSGNLEQLLLAVVK	
1704.9014	1704.9009	-0.0005	0	29	44	GLGTDEESILTLLTSR	
1749.8768	1749.8649	-0.0119	-7	245	259	SIPAYLAETLYYAMK	Oxidation (M)[14]
1802.8629	1802.8699	0.007	4	212	226	YMTISGFQIEETIDR	
1818.8578	1818.8562	-0.0016	-1	212	226	YMTISGFQIEETIDR	Oxidation (M)[2]
2658.2529	2658.2573	0.0044	2	161	185	DPDAGIDEAQVEQDAQALFQAGEL K	
2888.238	2888.2375	-0.0005	0	126	150	QVYEEEYGSSLEDDVVGDTSGYYQ R	

Spot #47. MALDI MS data - 41% coverage

**Gel Idx/Pos**	111/E21	**Instr./Gel Origin**	gloin/Spotset041117	**Process Status**	Analysis Succeeded
**Plate [#] Name**	[1] Sandrine	**Instrument Sample Name**		**Spectra**	1

Rank	Protein Name	Accession No.	Protein Score	Protein Score C. I. %	Total Ion C. I. %	Protein MW	Protein PI	Pep. Count	Intensity Matched
1	(P82979) Nuclear protein Hcc-1 (Proliferation associated cytokine-inducible protein CIP29)	HCC1_HUMAN	82	99.991		23582.4	6.13	9	10.974

Peptide Information

Calc. Mass	Obsrv. Mass	± da	± ppm	Start Seq.	End Seq.	Sequence	Modification
776.3719	776.3733	0.0014	2	17	22	QECLAR	Carbamidomethyl (C)[3]
781.4315	781.4313	-0.0002	0	31	36	QDLIHR	
832.441	832.4406	-0.0004	0	80	87	TVDVAAEK	
935.5196	935.512	-0.0076	-8	126	134	FGISSVPTK	
1069.5524	1069.5413	-0.0111	-10	1	9	ATETVELHK	N-Acetyl (Protein)[0]
1079.5844	1079.5908	0.0064	6	156	165	FGLNVSSISR	
1119.6045	1119.5909	-0.0136	-12	109	118	FNVPVSLESK	
1173.611	1173.6073	-0.0037	-3	92	101	ITSEIPQTER	
1871.8868	1871.8868	0	0	180	198	FGIVTSSAGTGTTEDTEAK	

Spot #48. no ID

Spot #49. MALDI MS data - 69% coverage

**Gel Idx/Pos**	112/E23	**Instr./Gel Origin**	gloin/Spotset041117	**Process Status**	Analysis Succeeded
**Plate [#] Name**	[1] Sandrine	**Instrument Sample Name**		**Spectra**	1

Rank	Protein Name	Accession No.	Protein Score	Protein Score C. I. %	Total Ion C. I. %	Protein MW	Protein PI	Pep. Count	Intensity Matched
1	(P63244) Guanine nucleotide-binding protein beta subunit 2-like 1 (Guanine nucleotide-binding prote	GBLP_HUMAN	197	100		35510.7	7.6	17	31.98

Peptide Information

Calc. Mass	Obsrv. Mass	± da	± ppm	Start Seq.	End Seq.	Sequence	Modification
746.4155	746.4218	0.0063	8	119	125	QIVSGSR	
829.5029	829.4927	-0.0102	-12	265	271	IIVDELK	
860.4512	860.45	-0.0012	-1	258	264	IWDLEGK	
920.4506	920.4496	-0.001	-1	2	8	TEQMTLR	N-Acetyl (Protein)[0]
936.4455	936.4401	-0.0054	-6	2	8	TEQMTLR	N-Acetyl (Protein)[0], Oxidation (M)[4]
1004.4982	1004.4901	-0.0081	-8	176	183	VWNLANCK	Carbamidomethyl (C)[7]
1059.5946	1059.5894	-0.0052	-5	309	317	VWQVTIGTR	
1090.5714	1090.5586	-0.0128	-12	131	139	LWNTLGVCK	Carbamidomethyl (C)[8]
1192.5593	1192.5509	-0.0084	-7	48	57	DETNYGIPQR	
1264.6532	1264.639	-0.0142	-11	89	99	LWDLTTGTTTR	
1309.6383	1309.6245	-0.0138	-11	107	118	DVLSVAFSSDNR	
1366.6824	1366.6626	-0.0198	-14	246	257	YWLCAATGPSIK	Carbamidomethyl (C)[4]
1476.6787	1476.6621	-0.0166	-11	213	225	DGQAMLWDLNEGK	
1492.6737	1492.657	-0.0167	-11	213	225	DGQAMLWDLNEGK	Oxidation (M)[5]
1907.8956	1907.8744	-0.0212	-11	156	172	FSPNSSNPIIVSCGWDK	Carbamidomethyl (C)[13]
1981.8708	1981.8475	-0.0233	-12	140	155	YTVQDESHSEWVSCVR	Carbamidomethyl (C)[14]
2276.1128	2276.0898	-0.023	-10	226	245	HLYTLDGGDIINALCFSPNR	Carbamidomethyl (C)[15]
2743.3103	2743.2932	-0.0171	-6	186	212	TNHIGHTGYLNTVTVSPDGSLCASG GK	Carbamidomethyl (C)[22]
3068.4419	3068.4177	-0.0242	-8	281	308	AEPPQCTSLAWSADGQTLFAGYTD NLVR	Carbamidomethyl (C)[6]

Spot #50. MALDI MS data - 46% coverage

**Gel Idx/Pos**	125/F1	**Instr./Gel Origin**	gloin/Spotset041117	**Process Status**	Analysis Succeeded
**Plate [#] Name**	[1] Sandrine	**Instrument Sample Name**		**Spectra**	1

Rank	Protein Name	Accession No.	Protein Score	Protein Score C. I. %	Total Ion C. I. %	Protein MW	Protein PI	Pep. Count	Intensity Matched
1	(P25788) Proteasome subunit alpha type 3 (EC 3.4.25.1) (Proteasome component C8) (Macropain subunit	PSA3_HUMAN	106	100		28512.1	5.19	11	21.053

Peptide Information

Calc. Mass	Obsrv. Mass	± da	± ppm	Start Seq.	End Seq.	Sequence	Modification
745.4202	745.4218	0.0016	2	86	92	SLADIAR	
763.4097	763.4136	0.0039	5	66	71	LFNVDR	
852.3846	852.3885	0.0039	5	93	99	EEASNFR	
949.4988	949.4862	-0.0126	-13	43	51	DGVVFGVEK	
1114.5602	1114.5524	-0.0078	-7	20	28	VFQVEYAMK	
1130.5551	1130.5471	-0.008	-7	20	28	VFQVEYAMK	Oxidation (M)[8]
1152.6047	1152.5947	-0.01	-9	100	109	SNFGYNIPLK	
1217.6484	1217.6427	-0.0057	-5	29	40	AVENSSTAIGIR	
1228.6936	1228.6848	-0.0088	-7	196	205	IIYIVHDEVK	
1380.7416	1380.7284	-0.0132	-10	72	85	HVGMAVAGLLADAR	
1396.7365	1396.728	-0.0085	-6	72	85	HVGMAVAGLLADAR	Oxidation (M)[4]
1820.9177	1820.9108	-0.0069	-4	208	223	AFELELSWVGELTNGR	
1917.8824	1917.9255	0.0431	22	1	19	SSIGTGYDLSASTFSPDGR	
1959.8931	1959.8827	-0.0104	-5	1	19	SSIGTGYDLSASTFSPDGR	N-Acetyl (Protein)[0]

Spot #51. MALDI MS data - 46% coverage

**Gel Idx/Pos**	30/B7	**Instr./Gel Origin**	gloin/Spotset050705	**Process Status**	Analysis Succeeded
**Plate [#] Name**	[1] Sandrine	**Instrument Sample Name**		**Spectra**	1

Rank	Protein Name	Accession No.	Protein Score	Protein Score C. I. %	Total Ion C. I. %	Protein MW	Protein PI	Pep. Count	Intensity Matched
1	(P30040) Endoplasmic reticulum protein ERp29 precursor (ERp31) (ERp28)	ERP29_HUMAN	114	100		29032.2	6.77	10	10.431

Peptide Information

Calc. Mass	Obsrv. Mass	± da	± ppm	Start Seq.	End Seq.	Sequence	Modification
937.4777	937.473	-0.0047	-5	198	204	WAEQYLK	
963.4815	963.4681	-0.0134	-14	100	107	LNMELSEK	
979.4764	979.4755	-0.0009	-1	100	107	LNMELSEK	Oxidation (M)[3]
1075.5378	1075.5292	-0.0086	-8	183	192	QGQDNLSSVK	
1134.6517	1134.6362	-0.0155	-14	244	253	SLNILTAFQK	
1247.5579	1247.5453	-0.0126	-10	60	69	FDTQYPYGEK	
1320.6622	1320.66	-0.0022	-2	113	122	ESYPVFYLFR	
1324.7147	1324.705	-0.0097	-7	37	48	GALPLDTVTFYK	
1608.754	1608.7411	-0.0129	-8	123	137	DGDFENPVPYTGAVK	
1708.7847	1708.7758	-0.0089	-5	209	223	ILDQGEDFPASEMTR	
1724.7797	1724.769	-0.0107	-6	209	223	ILDQGEDFPASEMTR	Oxidation (M)[13]
2468.1675	2468.1733	0.0058	2	76	99	LAENSASSDDLLVAEVGISDYGDK	

Spot #52. ESI MS/MS data

PA1B3_HUMAN Mass: 25832 Score: 228 Queries matched: 7
(Q15102) Platelet-activating factor acetylhydrolase IB gamma subunit (EC 3.1.1.47) (PAF acetylhydro

Check to include this hit in error tolerant search or archive report

Query	Observed	Mr(expt)	Mr(calc)	Delta	Miss	Score	Expect	Rank	Peptide
77	429,24	856,46	856,48	-0,02	0	39	0,036	1	QVNELVR
78	429,24	856,46	856,48	-0,02	0	-37	0,053	1	QVNELVR
105	459,75	917,5	917,48	0,01	0	16	5,6	1	GQHPNPLR
119	483,25	964,48	964,48	0	0	41	0,018	1	LGYTPVCR
150	521,31	1040,6	1040,6	0,01	0	42	0,012	1	AIVQLVNER
205	634,83	1267,65	1267,62	0,04	0	44	0,0078	1	PTPVQDVQGDGR
223	737,36	2209,05	2209,02	0,03	1	50	0,0014	1	SGEENPASKPTPVQDVQGDGR + N-Acetyl

Spot #53. MALDI MS data - 65% coverage

**Gel Idx/Pos**	54/C7	**Instr./Gel Origin**	gloin/Spotset050705	**Process Status**	Analysis Succeeded
**Plate [#] Name**	[1] Sandrine	**Instrument Sample Name**		**Spectra**	1

Rank	Protein Name	Accession No.	Protein Score	Protein Score C. I. %	Total Ion C. I. %	Protein MW	Protein PI	Pep. Count	Intensity Matched
1	(P60174) Triosephosphate isomerase (EC 5.3.1.1) (TIM) (Triose-phosphate isomerase)	TPIS_HUMAN	173	100		26806.8	6.51	13	16.144

Peptide Information

Calc. Mass	Obsrv. Mass	± da	± ppm	Start Seq.	End Seq.	Sequence	Modification
758.4406	758.4344	-0.0062	-8	149	155	VIADNVK	
850.4668	850.4597	-0.0071	-8	142	148	VVFEQTK	
954.4832	954.48	-0.0032	-3	6	13	FFVGGNWK	
1137.5721	1137.5609	-0.0112	-10	59	68	IAVAAQNCYK	Carbamidomethyl (C)[8]
1234.6022	1234.5957	-0.0065	-5	194	205	SNVSDAVAQSTR	
1326.6722	1326.6593	-0.0129	-10	206	218	IIYGGSVTGATCK	Carbamidomethyl (C)[12]
1414.79	1414.774	-0.016	-11	19	32	QSLGELIGTLNAAK	
1458.7223	1458.7159	-0.0064	-4	100	112	HVFGESDELIGQK	
1466.7234	1466.7153	-0.0081	-6	175	187	TATPQQAQEVHEK	
1586.738	1586.7401	0.0021	1	85	98	DCGATWVVLGHSER	Carbamidomethyl (C)[2]
1602.8889	1602.8835	-0.0054	-3	160	174	VVLAYEPVWAIGTGK	
1637.8203	1637.8057	-0.0146	-9	69	84	VTNGAFTGEISPGMIK	Oxidation (M)[14]
2192.0693	2192.0745	0.0052	2	33	52	VPADTEVVCAPPTAYIDFAR	Carbamidomethyl (C)[9]

Spot #54. MALDI MS data - 65% coverage

**Gel Idx/Pos**	158/G19	**Instr./Gel Origin**	gloin/Spotset040318	**Process Status**	Analysis Succeeded
**Plate [#] Name**	[1] Sandrine	**Instrument Sample Name**		**Spectra**	1

Rank	Protein Name	Accession No.	Protein Score	Protein Score C. I. %	Total Ion C. I. %	Protein MW	Protein PI	Pep. Count	Intensity Matched
1	(P51149) Ras-related protein Rab-7	RAB7_HUMAN	141	100		23759.9	6.4	13	3.435

Peptide Information

Calc. Mass	Obsrv. Mass	± da	± ppm	Start Seq.	End Seq.	Sequence	Modification
759.3995	759.4128	0.0133	18	127	132	IDLENR	
777.3889	777.3799	-0.009	-12	98	103	TLDSWR	
786.378	786.3712	-0.0068	-9	33	38	FSNQYK	
1013.4509	1013.4365	-0.0144	-14	139	146	AQAWCYSK	Carbamidomethyl (C)[5]
1036.5674	1036.5592	-0.0082	-8	39	48	ATIGADFLTK	
1057.6251	1057.6141	-0.011	-10	11	21	VIILGDSGVGK	
1175.6055	1175.6016	-0.0039	-3	104	113	DEFLIQASPR	
1187.6207	1187.6085	-0.0122	-10	70	79	FQSLGVAFYR	
1283.6266	1283.6165	-0.0101	-8	147	157	NNIPYFETSAK	
1475.7528	1475.7662	0.0134	9	114	126	DPENFPFVVLGNK	
1589.8281	1589.833	0.0049	3	158	171	EAINVEQAFQTIAR	
1663.8108	1663.8182	0.0074	4	56	69	LVTMQIWDTAGQER	Oxidation (M)[4]
1964.9487	1964.9669	0.0182	9	176	191	QETEVELYNEFPEPIK	

Spot #55. MALDI MS data - 23% coverage

**Gel Idx/Pos**	24/A20	**Instr./Gel Origin**	gloin/Spotset050705	**Process Status**	Analysis Succeeded
**Plate [#] Name**	[1] Sandrine	**Instrument Sample Name**		**Spectra**	1

Rank	Protein Name	Accession No.	Protein Score	Protein Score C. I. %	Total Ion C. I. %	Protein MW	Protein PI	Pep. Count	Intensity Matched
1	(P28072) Proteasome subunit beta type 6 precursor (EC 3.4.25.1) (Proteasome delta chain) (Macropain	PSB6_HUMAN	71	99.895		25569.5	4.8	7	16.642

Peptide Information

Calc. Mass	Obsrv. Mass	± da	± ppm	Start Seq.	End Seq.	Sequence	Modification
755.3327	755.336	0.0033	4	75	79	IFCCR	Carbamidomethyl (C)[3,4]
758.296	758.3068	0.0108	14	119	123	EMCYR	Carbamidomethyl (C)[3]
774.2909	774.2886	-0.0023	-3	119	123	EMCYR	Carbamidomethyl (C)[3], Oxidation (M)[2]
847.4268	847.4296	0.0028	3	201	209	DGSSGGVIR	
851.4733	851.4763	0.003	4	68	74	LTPIHDR	
1083.543	1083.5415	-0.0015	-1	54	63	TTTGSYIANR	
1110.6517	1110.6353	-0.0164	-15	221	230	QVLLGDQIPK	
1115.6055	1115.5997	-0.0058	-5	210	220	LAAIAESGVER	
